# Control of Protonated Schiff Base Excited State Decay within Visual Protein Mimics: A Unified Model for Retinal Chromophores

**DOI:** 10.1002/chem.202102383

**Published:** 2021-10-28

**Authors:** Baptiste Demoulin, Margherita Maiuri, Tetyana Berbasova, James H. Geiger, Babak Borhan, Marco Garavelli, Giulio Cerullo, Ivan Rivalta

**Affiliations:** a Laboratoire de Chimie Univ Lyon, Ens de Lyon, CNRS UMR 5182 Université Claude Bernard Lyon 1 69342, Lyon (France); b IFN-CNR, Dipartimento di Fisica Politecnico di Milano Piazza Leonardo da Vinci 32, I-20133 Milano (Italy); c Department of Chemistry Michigan State University East Lansing, MI 48824 (USA); d Dipartimento di Chimica Industriale “Toso Montanari” Università degli Studi di Bologna Viale del Risorgimento 4, 40136 Bologna (Italy)

**Keywords:** excited state dynamics, QM/MM methods, retinal Schiff base, rhodopsin mimics, ultrafast optical spectroscopy

## Abstract

Artificial biomimetic chromophore-protein complexes inspired by natural visual pigments can feature color tunability across the full visible spectrum. However, control of excited state dynamics of the retinal chromophore, which is of paramount importance for technological applications, is lacking due to its complex and subtle photophysics/photochemistry. Here, ultrafast transient absorption spectroscopy and quantum mechanics/molecular mechanics simulations are combined for the study of highly tunable rhodopsin mimics, as compared to retinal chromophores in solution. Conical intersections and transient fluorescent intermediates are identified with atomistic resolution, providing unambiguous assignment of their ultrafast excited state absorption features. The results point out that the electrostatic environment of the chromophore, modified by protein point mutations, affects its excited state properties allowing control of its photophysics with same power of chemical modifications of the chromophore. The complex nature of such fine control is a fundamental knowledge for the design of bio-mimetic opto-electronic and photonic devices.

A joint experimental-computational study elucidates the photophysics of retinal Schiff bases embedded in artificial proteins capable of mimicking the color tunability of natural visual pigments as compared to solvated chromophores. Combining ultrafast transient absorption spectroscopy with quantum mechanics/molecular mechanics simulations allowed monitoring nonlinear optical signals of the chromophore, shedding light on the complexity beyond the fine control of its excited state lifetime.

## Graphical Abstract

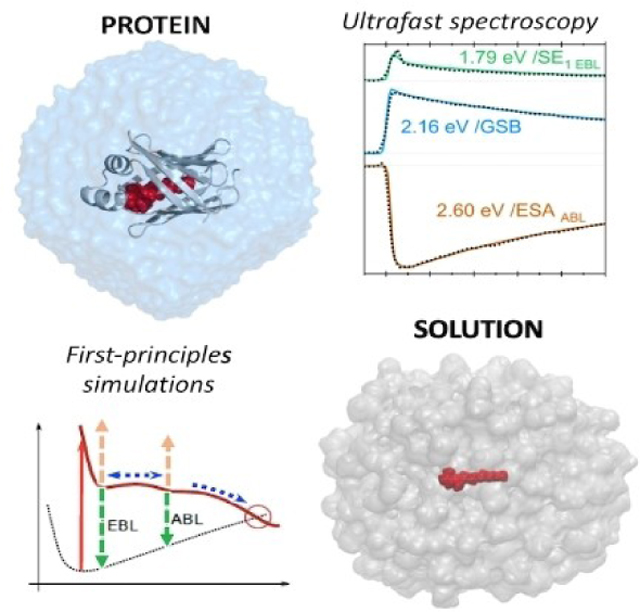

## Introduction

The abundance of distinct rhodopsin-like proteins occurring in Nature, and the variety of their properties, activities and functions, make them interesting candidates for the design of tunable photo-activated bio-inspired nanodevices, which could be employed for opto-mechanical energy transduction^[1]^ and in opto-electronics or opto-genetics.^[2]^ Artificial tuning of natural rhodopsins has already been successfully achieved, leading to fundamentally different biological functions. Single point mutations of the binding pocket have been used to convert the inward proton pumping bacteriorhodopsin into an outward chloride pump,^[3]^ to allow interconversion between chloride, sodium and proton pumps in bacterio- and halorhodopsins,^[4]^ to create inward proton pumps^[5]^ and to transform ion pumps into ion gated channels.^[6]^

Design of new rhodopsin-based devices that can achieve the full control of photophysical and photochemical properties requires an accurate understanding of the interactions between the protein binding pocket and the bound retinal Schiff Base (SB). Indeed, the chromophore is highly sensitive to modifications of the protein backbone, via either electrostatic interactions or steric constraints, due to the characteristic intramolecular charge transfer (CT) character of its spectroscopic (S_1_) excited state.^[7]^ The presence of a counterion for the protonated Schiff base (PSB) formed upon retinal binding affects the relative energies of the PSB ground and excited states, leading to significant spectral shift of the corresponding vertical absorption energy.^[8]^ Moreover, the spectroscopic electronic transition (associated to a one-electron HOMO(H)→LUMO(L) transition) is further tuned by the localization of the electrostatic potential along the PSB polyene chain, resulting in absorptions maxima ranging from 420 nm (in human short wave sensitive pigment)^[9]^ to 587 nm (in sensory rhodopsin I).^[10]^ Combined experimental and theoretical studies on gas-phase PSBs have demonstrated that an evenly distributed electrostatic field can lead to significant red-shift of the absorption maximum.^[11]^

**Figure 1.**
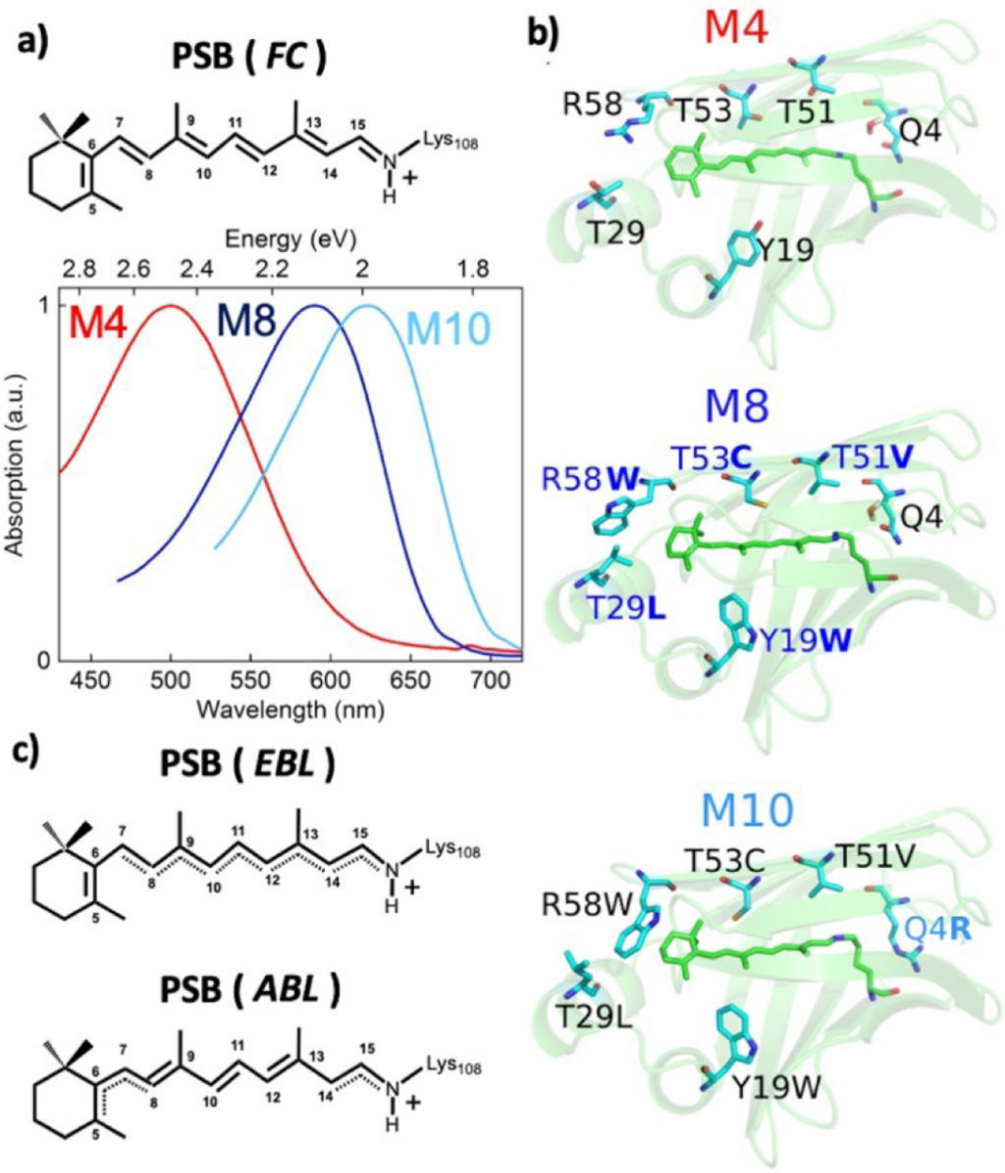
a) Sketch of the retinal all-*trans* protonated Schiff-base (PSB) covalently bound to Lys108 (K108) of the hCRBPII rhodopsin mimics, including atom numbering, at the Franck-Condon (FC) region and normalized absorption spectra for three selected mimics, labelled as M4, M8 and M10. b) X-ray structures of the selected mimics, highlighting the residues in M4 that undergo mutations in M8 and subsequently in M10. c) Transient fluorescent intermediates of PSB along the S_1_ potential energy surface (PES), featuring C–C even bond lengths (EBL) and alternated bond lengths (ABL).

Obtaining systems for which the electrostatic potential around the PSB can be accurately tuned is of crucial importance for improving our current models and understanding of these interactions. Recently, protein systems based on cellular retinoic acid binding protein type II (CRABPII) and human cellular retinol binding protein type II (hCRBPII) have been proposed, in which the local environment of the aldehyde site of an embedded all-*trans* retinol or retinoic acid molecule has been mutated in order to allow formation of a PSB, thus mimicking natural opsin proteins.^[12]^ Binding pocket mutations were carefully targeted to improve encapsulation of the retinal molecule, which is important for effective control of spectral tuning.^[13]^ Some of the authors recently proved that hCRBPII is capable of covalently binding a retinal molecule (see Figure 1) upon two point mutations at the PSB binding site (Q108 K and K40 L). Few (6–7) point mutations inside the Q108 K:K40 L:hCRBPII binding pocket lead to complete retinal encapsulation and spectacular protein color tunability, allowing the design of eleven rhodopsin mimics (M1-M11) with absorption maxima spanning the entire visible spectrum, from 425 to 644 nm (i.e. from 1.9 to 2.9 eV).^[14]^ It is worth noting that the hCRBPII mimics do not contain a negatively charged counterion associated with the retinal PSB (see Figure 1), contrary to natural opsins, demonstrating how original electrostatic control of PSB absorption properties can be artificially achieved.

This ground-breaking protein manipulation can pave the way to innovative retinal-based optical probes, especially if the tuning of the absorption properties can be accompanied by a rational control of the excited state decay and of the photochemical pathways. Advanced computational studies provide a route for fundamental understanding of ground and excited states properties.^[15]^ A first exploratory attempt to model and study artificial rhodopsin systems involved the CRABPII proteins (less tunable than hCRBPII), suggesting the population of two different excited states upon photo-excitation.^[16]^ Modeling of the absorption tuning in some of the hCRBPII mimics has been achieved by various groups^[17]^ by quantum mechanics (QM)/molecular mechanics (MM) simulations, quantifying some of the long-range effects determining the color tuning of hCRBPII proteins. Still, the mechanism governing the photophysical and photochemical response following light absorption at various wavelengths of these artificial proteins remains an open question. Direct comparison of ultrafast spectroscopy measurements with theoretical simulations can provide unique information on the excited state decay in these rhodopsin mimics.

Here, we report an experimental and theoretical study of photoinduced processes in hCRBPII mimics, combining ultrafast pump-probe spectroscopy with QM/MM models based on multi-reference wavefunctions. Our study is inspired by previous successful characterizations of the primary events of the photochemistry in visual rhodopsins^[18]^ and (more recently) solvated PSBs,^[19]^ where similar approaches combining QM/MM methods with pump-probe spectroscopy were implemented. It should be mentioned that our recent work on solvated PSBs^[19]^ addressed “static” studies of potential energy surface (PES) along both the bond relaxation and the torsional modes, being able to predict differences in PSB lifetimes obtained from semi-classical molecular dynamics simulations and ultrafast spectroscopy experiments. Here, we demonstrate, both experimentally and computationally, how protein design can critically induce electrostatic control of the excited state properties of the retinal chromophores. The obtained fundamental knowledge of the photoinduced response of PSBs under electrostatic control will be valuable for the design of bio-inspired optical devices.

For our investigations we selected three spectrally separated hCRBPII mimics, labelled M4, M8 and M10 (following reference^[14]^), primarily due to the availability of their crystal structures, as depicted in Figure 1b–c. M4 contains the minimal number of mutations that allow for the formation of the PSB (Q108 K and K40 L), with a binding pocket characterized by several polar residues (Y19, T29, T51, T53 and R58) surrounding the PSB and a water molecule bridging between the PSB iminium group and Gln4 (see Figure1).^[14]^ Multiple mutations lead to M8, i.e. Y19 W, T29 L, T51 V, T53 C and R58 W, which increase the hydrophobicity of the binding pocket by inserting apolar (tryptophan, leucine, and valine) residues. A single mutation is associated, instead, to the M8-to-M10 transition, involving Gln4 mutation into an arginine (Q4R), resulting in loss of the bridging water molecule in the active site and in the change of PSB iminium conformation from *cis* to *trans*, according to the crystal structure and like other mimics featuring a mutation of Gln4.^[14]^

## Methodology and Computational Details

### Sample preparation

The M4, M8 and M10 proteins were prepared according to the methods described in detail in ref.^[14]^ Briefly, the hCRBPII proteins were expressed from the pET17b vector. Plasmids encoding mutants M4, M8 and M10 were transformed into the E. coli BL21 (DE3)pLysS strain and selected on Amp (100ug/mL)/LB plates. A single colony was inoculated into LB/amp media to generate starter culture and then transferred to 1 L LB/Amp media. Cells grew at 37°C until OD600 reached 0.6 and hCRBPII expression was induced with addition of IPTG to a final concentration of 1 mM. Expression continued for 20 h at 23°C. The cells were harvested by centrifugation, resuspended in 10 mM Tris pH 8.0 containing DNase I (Roche), lysed by sonication, cleared from debris and loaded on the FastQ anion exchange resin. After two washes with Tris buffer, hCRBPII mutants were eluted with 200 mM NaCl, 10 mM Tris pH 8.0 buffer. Samples were desalted using an Ultrafiltration cell with MWCO 10,000 Da (Millipore). Samples were further purified using a BioLogic DuoFlow (BioRad) employing SOURCE–Q anion exchange resin (GE Health Sciences). Pure protein eluted at 80 mM NaCl was stored in the elution buffer containing 10% glycerol, and used as is to generate complexes with all-trans-PSB (Sigma). The complex was generated by incubation of the protein with 0.5 equivalents of retinal for two hours. Schiff base formation was verified using UV-vis absorption spectroscopy.

### Ultra-fast pump-probe spectroscopy

The experimental pump-probe apparatus is based on a regeneratively amplified Ti:sapphire laser (Coherent, Libra) producing 100 fs, 4 mJ pulses at 800 nm and 1 kHz repetition rate. A home-made optical parametric amplifier produces the pump pulses, with 10-nm bandwidth and ≈70-fs duration, tunable in the visible spectral region. A small fraction of the fundamental wavelength pulse is focused on a 2-mm-thick sapphire plate to generate a broadband single-filament white light continuum, spanning from 450 to 720 nm, acting as probe. The pump and probe pulses are synchronized by a motorized translation stage and spatially overlapped on the sample in a slightly non-collinear geometry. After the sample, the probe beam is focused onto the entrance slit of a high-resolution spectrometer (Acton, Princeton Instrument) equipped with fast electronics (Stresing Entwicklungsbüro), allowing single shot recording of the probe spectrum at the full 1 kHz repetition rate of the laser.^[20]^ By recording pump-on and pump-off probe spectra, we extract the differential transmission (ΔT/T) signal as a function of probe wavelength and pump-probe delay as ΔT/ T=(T_on–_T_off_)/T_off_. Our setup achieves sensitivity down to ≈10^−5^ at each probe wavelength. The temporal resolution (taken as full width at half-maximum of pump-probe cross-correlation) is estimated to be ≈100 fs over the entire probe spectrum. The pump fluence used in the series of experiments was kept ≈0.5 mJ/cm^2^. The samples have OD≈0.2 absorbance, measured in a 1 mm quartz cuvette. Global analysis of the 2D ΔT/T maps is performed using the Glotaran software.^[21]^

### Computational details

The hCRBPII crystal structures were taken out of the Protein Data Bank, under the codes 4EXZ, 4EFG and 4EEJ, for the M4, M8 and M10 systems respectively.^[14]^ These structures contain two monomers, but only one was kept for the computation by selecting the chain A or the chain where the PSB residue was complete. The full PSB residue (comprising the lysine 108 and the retinal side chain) was parameterized using the GAFF force field^[22]^ within the Antechamber software, available in the AmberTools 14 package.^[23]^ The protonation states have been assigned by the tLeap software, also available in AmberTools 14. The Amber ff99SB^[24]^ force field was used for all the standard protein residues. The structures were initially refined at the MM level with a steepest descent algorithm, all the heavy atoms being restrained to their crystallographic positions. A QM/MM scheme was then employed, where the PSB up to the Cɛ atom of the nearby bound lysine is treated at the QM level, while the rest of the protein is treated by a classical force field. The frontier between the QM and MM parts was capped with a single hydrogen atom, and the classical charges of the PSB atoms were redistributed over the atoms of the protein. We have used an electrostatic embedding scheme, which allows for the treatment of the electrostatic interactions between the MM and the QM layer at the QM level of theory. The PSB molecule was optimized at the multi-configurational complete active space (CASSCF) level,^[25]^ where the active space comprises its whole π system, giving 12 electrons in 12 orbitals. Three states of interest were included in the state-averaged computations (SA3-CASSCF) for ground state (GS) absorption computations, i.e. the first three singlet states S_0_, S_1_ and S_2_. The geometry optimizations were carried out at the SA3CASSCF level using the COBRAMM^[26]^ package, which interfaces MOLCAS^[27]^ for the QM computations of energies and gradients, the AMBER package for the treatment of the classical part and the geometry optimization algorithms of Gaussian 09.^[28]^ The ground and excited states energies were corrected by including a second order correction to the CASSCF wavefunction with the CASPT2^[29]^ method, in its single state (SS) and multi-state^[30]^ (MS) variants. To avoid the so-called intruder states, an imaginary shift of 0.2 has been set. The zero-order Hamiltonian shift was set to zero according to recently published results.^[31]^ The combined CASPT2// CASSCF/AMBER approach (hereafter CASPT2) has been shown to quantitatively interpret the photochemistry of PSB embedded in rhodopsins.^[32]^

It is expected that explicit solvent molecules are required for a realistic modeling of these systems, in order to account for the influence of the surrounding water arrangements and hydrogen bonding network (HBN) on the PSB intramolecular charge-transfer excited state properties. We have followed an approach that has been successfully used on similar (CRABPII) proteins by Huntress et al.^[16]^ The three proteins under investigation were solvated by using a 10 Å octahedral box of TIP3P water molecules and neutralized by adding Na^+^ ions. We then performed classical molecular dynamics (MD) to obtain solvent conformations around the proteins, using harmonic constraints (30 kcal/mol) on all the protein and PSB atoms. This is an efficient way to obtain water arrangements around the protein equilibrium structure, while assuming that the crystal structure is indeed a reasonable approximation of the protein equilibrium structure in solution. The minimized protein structures were heated to 300 K, and then equilibrated at 1 bar for 1 ns. After equilibration, a 100 ns trajectory was obtained for each protein using the GPU version of PMEMD^[33]^ as available in Amber 12.^[34]^ Out of these trajectories, ten snapshots (i.e. one every 10 ns) were selected to be further investigated with the QM/MM protocol already described above. Unless otherwise stated, the results reported in the following sections refer to the average values computed on top of ten snapshots for each rhodopsin mimic. For the excited state optimizations, the QM/MM scheme has been slightly modified to reduce the computational effort, with the full π system up to the Cɛ atom of the nearby lysine still belonging to the QM (High) layer, while the remaining part of the ionone ring and the lysine residues were put in a movable (medium) layer, treated at the MM level. The excited state optimizations were carried out including four states in the stateaveraged computations (SA4-CASSCF), in order to ensure the S_2_ state being included in the computations. The energies were computed at the SS- and MS-CASPT2 levels. The excited state absorptions (ESAs) were computed on top of all geometries using SA10-CASSCF wavefunctions, since these excitations arising from S_1_ reach high-lying states, and require inclusion of such excited states. The 6–31G* basis set was used for the geometry optimizations, while the larger ANO S basis set was used for computing all the energies on top of the optimized structures. The total cost of the computations reported in this work amount to ca. 24’000 CPU hours on specifically designed machines.

## Results and Discussion

Figure 1 shows that the selected hCRBPII proteins have distinct experimental absorption maxima at ca. 490, 590 and 623 nm, corresponding to ca. 2.48, 2.1 and 1.99 eV, for M4, M8 and M10, respectively. The absorption bandwidth (calculated as FWHM) is broader for M4 (0.73 eV) than for M8 (0.43 eV) and M10 (0.40 eV). Table 1 reports the vertical excitation energies computed for the three mimics at the multi-configurational complete active space (CASSCF)^[25b]^ level corrected with the single-state (SS) CASPT2^[29]^ method on top of the optimized GS geometry, i.e. in the Franck-Condon (FC) region. These energies, reported as averages of the various conformations extracted from the molecular dynamics sampling (see Supporting Information for further details), are in good agreement with the experimental absorption maxima values, displaying differences of 0.19, 0.03 and 0.01 eV for M4, M8 and M10, respectively. The agreement of CASPT2 computed vertical excitations and experimental absorption maxima in hCRBPII pigments is in line with what observed in animal rhodopsins.^[35]^ The values obtained at the multi-state (MS)^[30]^ MS-CASPT2 level give a slightly worse agreement, apart from M4. This can be explained by considering the high configuration mixing in the excited state manifold obtained in the M4 case (see Table S1 in Supporting Information), where the S_1_ and S_2_ states feature mixing between the single H→L and the double (H→L)^2^ excitations. Thus, we believe that for the M4 case, the multistate treatment provides an appropriate description of the S_1_/S_2_ states and more reliable transition energies than SS-CASPT2. This mixing does not appear in M10. The M8 case, instead, showed two distinct behaviors (M8’ and M8’’) among the ten solvent configurations selected from the molecular dynamics sampling, as discussed in the Supporting Information. Hereafter, we will only consider M8 configurations with appropriate description of the S_1_ state and transition energies in agreement with experimental data, namely the M8’ subset.

In contrast to M4, the experimental absorption maxima of M8 and M10 are very close to those computed^[11]^ and measured^[29a]^ for PSBs in gas phase (see Supporting Information and Table S7), indicating that in these proteins the PSB is more effectively shielded from the solvent. This should be expected considering that several M4 polar residues are mutated to hydrophobic in M8 and M10 (see Figure 1c). The gas-phase excitation energies of PSBs extracted from the various proteins are all in the range 1.98–2.17 eV at SS-CASPT2 level, indicating that the PSB’s geometry plays a minor role and spectral tuning is mainly due to the solvated protein scaffold, with the mutation of hydrophobic residues in M8/M10 with respect to M4 justifying the spectral red-shift. Still, the standard deviations of the excitation energies due to the different selected configurations of the explicit solvent molecules vary among the various proteins, demonstrating the importance of including such statistics in the modeling.

Notably, the S_0_→S_1_ transition energies computed for the various solvent configurations in M4 are much more scattered compared to M10, indicating a larger influence of the conformation of the hydrogen bonding network in M4, and in good agreement with the trends of the experimental linear absorption bandwidths. The computed difference in permanent dipole moments (Δμ) between S_0_ and S_1_ indicates a more effective charge transfer in M10 and M8 than in M4 (see Table 1) upon excitation to S_1_, as corroborated by the Mulliken charge analysis, where the PSB is separated in two fragments at the C_11–_C_12_ bond (see Figure 1a), showing that only 27% of the charge is transferred from the PSB-Lysine108 to the ionone ring side of the molecule in M4, while *>*45% is transferred in M8 and M10. This is in line with the larger PSB exposure to solvent molecules in M4 than in M8-M10. Notably, such behavior of the PSB in M4 is quite close to that recently reported for QM/MM all-*trans* PSB models in methanol solutions.^[19]^ Thus, the protein scaffold plays a crucial role in determining the optical absorption of the mutants. In particular, the difference in absolute QM energies of PSB computed in the solvated proteins and in gas phase, i.e. the energy stabilization due to the electrostatic interactions with the environment, shows that the S_1_ state in M4 is less stabilized than S_0_ and S_2_ states, leading to the observed blue-shift. On the contrary, the environment similarly affects all states in M8 and M10, again proving the large protein scaffold shielding in these cases (Figure S7).

The presence of two possible transient fluorescent intermediates (TFI) along the S_1_ PES, corresponding to two intermediate geometries along the structural relaxation on S1, has been previously reported for PSBs in solution^[19,36]^ and embedded in CRABPII proteins.^[16]^ These two S_1_ structures feature distinct stimulated emission (SE) wavelengths and are characterized by differences in the C–C bond length alternation (BLA), defined as the difference between the average distances of single (~1.45 Å) and double (~1.35 Å) bonds in the PSB polyene chain. BLA close to zero indicate the presence of even bond lengths (EBL) and values close to 0.1 Å correspond to fully alternated bond lengths (ABL). Thus, we have explored the S_1_ PES of the selected hCRBPII proteins as function of PSB’s BLA.

For M4, all geometry optimizations starting from the GS minimum geometry yielded EBL structures for S_1_, with BLA ~0, opposite to M10 for which all S_1_ optimized geometries featured ABL in the central region of the PSB molecule, with overall BLA ~0.06 Å. Consistently, similar BLA values are found in the M8’ and M8’’ configurations, with the M8’ configurations being always associated to ABL structures. Focusing on specific variations of C–C bonds upon photoexcitation (see Figure S8-S10 in the Supporting Information), we found that in M4 the EBL geometries still show a preferential elongation of the C_13–_C_14_ bond (from 1.363 Å in S_0_ to 1.447 Å in the S_1_ on average), as in the bacteriorhodopsin (bR).^[37]^ Notably, the same occurs for M8’ (EBL) and for M8’’ (ABL), where the C_13–_C_14_ bond still results slightly more elongated than the C_11–_C_12_ bond. On the contrary, all M10 (ABL) structures display a larger elongation of the C_11_ C_12_ bond (from 1.357 Å in the S_0_ state to 1.466 Å in the S_1_ state) compared to the others. This result indicates how the binding site Q4R mutation in proximity of Lys108 affects the PSB excited state geometry in M10.

The PSB photoisomerization (in gas phase, solution and proteins) follows a three-mode pathway,^[18b,38]^ where the first two modes involve rotation around the reactive bond upon activation of the C–C stretching mode and dominate the excited state decay up to the conical intersection (CI) seam.^[18b]^ The third, hydrogen out-of-plane mode is known to control the efficiency of the (forward vs. reverse) photoisomerization,^[39]^ providing a dynamical control of the photoproduct and will not be considered here. Ultrafast photoisomerization reactions, such as that occurring in rhodopsin within 200 fs, are not affected by the interplay between the (covalent) S_2_ state and the spectroscopic (ionic) S_1_ state, and therefore follow the so-called *two-state* model.^[40]^

**Figure 2.**
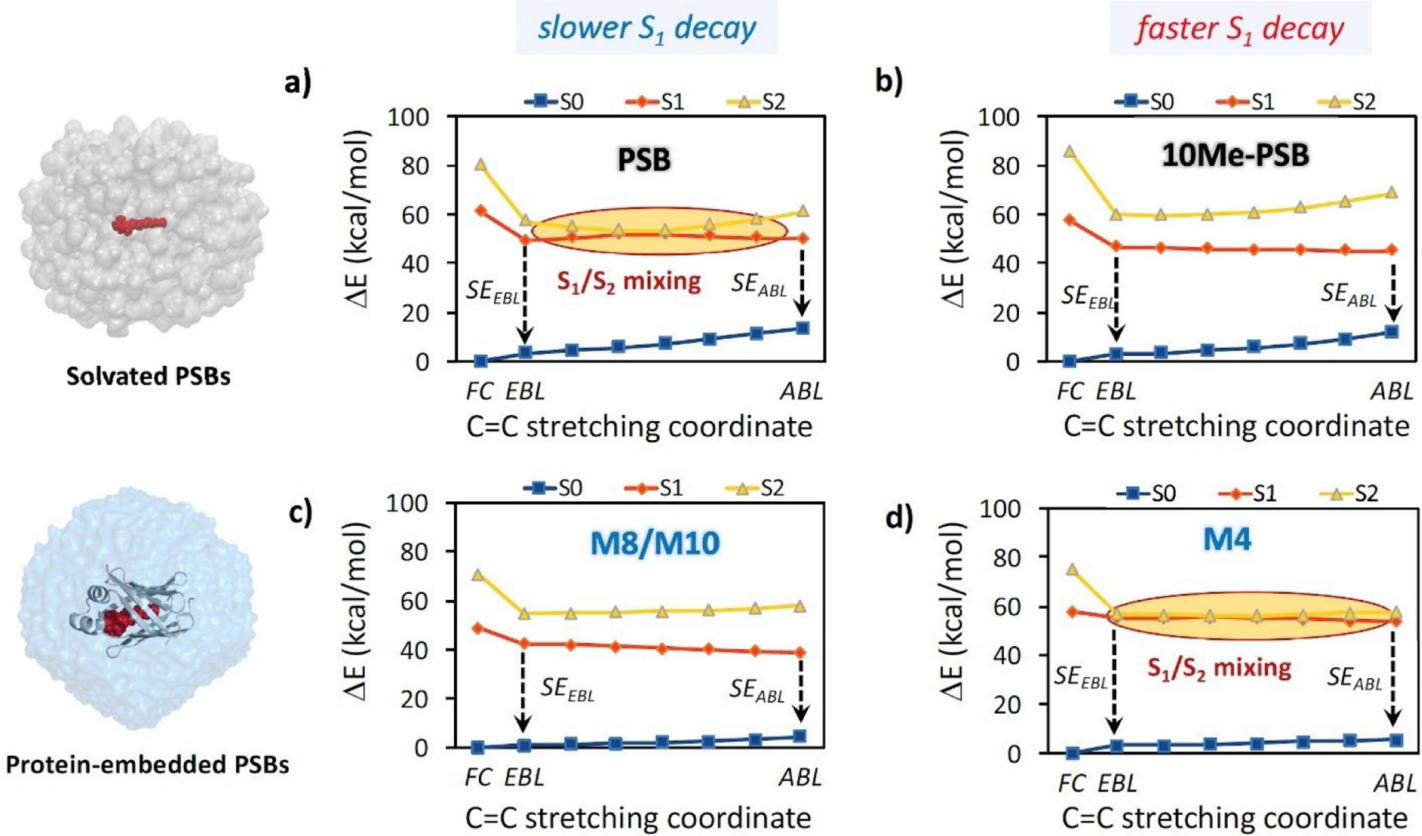
Minimum energy pathways from the FC region to EBL and ABL geometries, comparing solvated all-*trans* PSB (a) and 10-Methylated all-*trans* PSB (b), reproduced from ^[19]^, and all-*trans* PSB embedded in the M10 (c, with M8 showing very similar profile) and M4 (d) proteins. A single representative configuration is reported for clarity while the complete set of data is reported in the Supporting Information. The plotted energies are calculated at the SS-CASPT2 level. Regions featuring S_1_/S_2_ mixing are highlighted with shaded ellipses and stimulated emission (SE) processes from the EBL and ABL transient fluorescent configurations are indicated with arrows.

In contrast, slower photoreactions, such as those occurring in bR (500 fs) or in solvated PSB (*>*1 ps), follow a *three-state* mechanism.^[19,41]^ Indeed, we have recently shown how an avoided S_1_/S_2_ crossing along the EBL→ABL minimum energy path (MEP) contributes, along with unfavorable topography of the S_1_/S_0_ CI region, to a decrease in the photoisomerization rate (~4 ps) ^1^ of solvated all-*trans* PSB.^[19]^ Moreover, minimal chemical modifications of the PSB backbone (i.e. a methyl substitution at C_10_, namely 10Me-PSB) can significantly increase the charge-transfer character of the S_1_ state, enlarging the S_2_/S_1_ energy gap and thus contributing to a speed-up of the photoinduced decay. Here, we evaluate the *three-state* mechanism in the selected hCRBPII proteins, monitoring the mutation-induced electrostatic effects along the C–C stretching mode. Figure 2 shows the MEPs connecting (by constrained scan of linearly interpolated structures) the EBL and ABL geometries computed at the QM/MM SA3-CASSCF//SS-CASPT2/6–31G* level for representative configurations of the M4, M8 and M10 mimics (all computed MEPs are reported in the Supporting Information, see Figure S11–S13) and the comparison with solvated PSBs.

In M4 (Figure 2d), the transition from EBL to ABL in the S_1_ state encounters an avoided crossing with the S_2_ state, leading to an average energy barrier of 1.65 kcal/mol, explaining why all S_1_ geometry optimizations yielded an EBL geometry. Along this MEP, S_1_ and S_2_ state wavefunctions are highly mixed, featuring contributions from H→L, (H→L)^2^ and H→L+1 configurations. As shown in Figure 2d, the behavior of M4 is analogous to that of solvated PSB, with avoided crossing along the MEP and significant S_1_/S_2_ mixing. On the other hand, the avoided crossing is not found for the EBL!ABL MEPs of M8’ and M10, due to the increase of the S_2_/S_1_ energy gap that is present already in the FC region and is maintained along the C–C stretching coordinate, analogous to 10Me-PSB. For these mimics, the S_1_ PESs result slightly downhill (almost flat) for the EBL to ABL transition. These results go along with the charge transfer characters of the S_0_→S_1_ transition reported in Table 1 for M4 (weak CT) and M8/M10 (larger CT) and those found for solvated PSBs,^[19]^ showing how the electrostatic effects of mutations affecting the charge transfer of the spectroscopic state in turn operate on the interactions between covalent and ionic (i.e. S_2_ and S_1_) states and affect the excited state relaxation pathways. The resulting electrostatic effect, thus, parallels the effect of chemical modification of the PSB backbone,^[19]^ showing how the control of the intramolecular charge transfer can be achieved by either local (chemical) or environmental (electro-static) perturbations with analogous efficacy. However, as indicated in Figure 2 and discussed below, the picture resulting from the C–C bond relaxation MEPs fits the trend of experimental S_1_ lifetimes for solvated PSBs (faster S_1_ decay for 10Me-PSB with respect to PSB) but contrasts with that of hCRBPII pigments.

**Figure 3.**
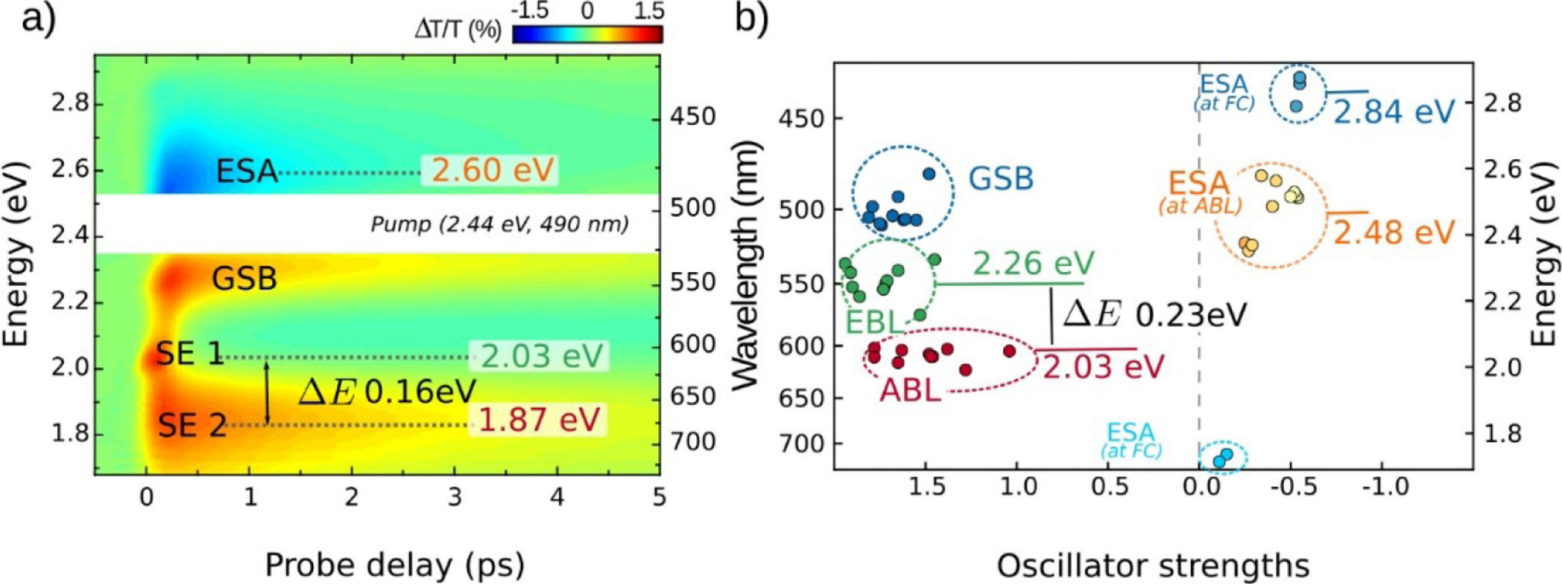
Ultrafast pump-probe spectroscopy of M4. (a) Experimental ΔT/T map. (b) Vertical excitation energies computed at the MS-CASPT2 level (with ANO–S basis set) on top of FC geometry (blue), EBL (green) and ABL (red and orange) geometries. Computed ESAs are shown with negative oscillator strengths to be distinguished from GSB and SE signals with positive oscillator strengths.

**Figure 4.**
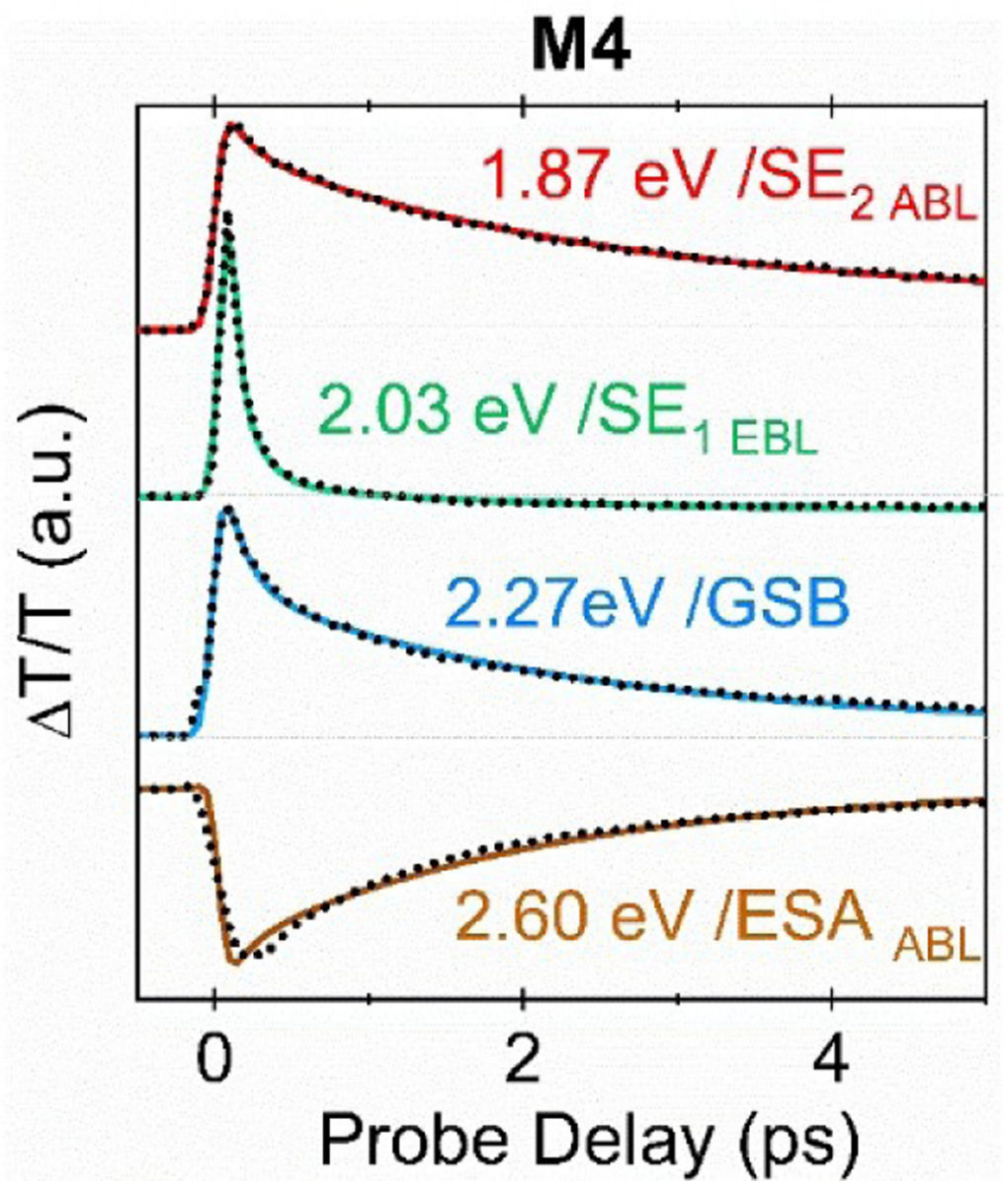
ΔT/T time traces at selected probe photon energies (dotted lines), representative of the GSB, SE_EBL_, SE_ABL_ and ESA_ABL_ spectral features of M4. Corresponding exponential fits are also reported (solid lines).

The characterization of the excited states PES suggests the presence of a rather small barrier (in M4) or barrierless (in M8 and M10) pathways along the C–C stretching mode, indicating that experimental detection of transient species would require ultrafast (sub-picosecond) optical spectroscopy. Figure 3 and Figure 4 show the experimental differential transmission (ΔT/T) signal maps (as a function of probe photon energy and pump-probe delay) and the ΔT/T time traces at selected probe photon energies, respectively, for M4, upon photo-excitation at 2.44 eV. The energy interval of ≈ 0.15 eV around the excitation energy was removed due to pump beam scattering. The ΔT/T data were subjected to global analysis and the corresponding decay associated spectra (DAS) are reported in Figure S18. Three positive bands are observed at 2.27, 2.03 and 1.87 eV respectively (Figure 3a). The 2.27 eV band is assigned to the residual red-tail of the GS bleaching (GSB) of the S_0_→S_1_ transition, peaking at 2.44 eV, while the bands at 2.03 and 1.87 eV correspond to SE from S_1_ to S_0_. A dual-peaked structure of the SE band is found, with a first peak centered around 2.03 eV (SE_1_) having a fast decay, with a time constant of 0.14±0.01 ps, while the GSB and the second SE (SE_2_) bands decay with a similar time constant of 2.10±0.01 ps (Figure 4). Moreover, a negative signal, corresponding to transitions from S_1_ to higher lying states (excited state absorptions, ESAs), is also present around 2.60 eV. The ESA signal has a similar temporal evolution as the GSB and decays with a time constant of 2.10±0.01 ps. Figure 3 shows comparison between the experimental ΔT/T signals (Figure 3a) and the computed S_0_→S_1_ and S_1_→S_n_ transitions obtained at different geometries along the S1 relaxation MEPs (Figure 3b), i.e. the FC geometry and the EBL and ABL transient structures, allowing specific assignments of the experimental peaks of M4. The S_1_→S_0_ transition energies computed at the EBL and ABL geometries are separated in energy by ca. 0.23 eV and can be gathered in two groups centered around 2.26 and 2.03 eV, respectively. Notably, the two experimental SE_1_ and SE_2_ bands centered at 2.03 and 1.87 eV, respectively, are separated by ca. 0.16 eV.

The computed energies are slightly, almost rigidly, blue-shifted (*<*0.25 eV discrepancy) with a qualitative agreement that allows us to assign the SE_1_ band to the emission from the EBL fluorescent state (SE_EBL_) and SE_2_ to emission from the ABL state (SE_ABL_). This theoretical assignment is supported by the experimental time evolution of these two bands (Figure 4), showing how the SE_EBL_ signal maximum is reached instantaneously (within the instrumental response function) with respect to the slightly delayed SE_ABL_ signal, indicating how the former is associated to a TFI (i.e. the EBL) that arises prior to the latter (i.e. associated to the ABL), as expected for excited state decay computed along the FC→EBL→ABL bond relaxation pathway. Our computations do not predict ESA signals (with opposite sign to SE) between 1.8 and 2.1 eV, excluding that the dual-peaked structure of the experimental SE band can be attributed to the presence of an overlapping ESA band in this region, as it has been observed for solvated PSBs.^[19,36]^ The assignment of the SE_1_ and SE_2_ bands to two different fluorescent states (SE_EBL_ and SE_ABL_) is further supported by their drastically different decay dynamics and by ESA signals associated to these TFIs. Indeed, our computations predict an ESA signal at around 2.48 eV associated to the ABL structure, in good agreement with the experimental signal recorded at 2.60 eV, which is then assigned as ESA_ABL_. Notably, the time evolution of this ESA_ABL_ peak appears to be similar to that of the SE_2_ peak centered at 1.87 eV (see Figure 4), corroborating the theoretical prediction. The ultrafast decay of the SE_EBL_ band is confirmed by the first DAS in Figure S18a.

**Figure 5.**
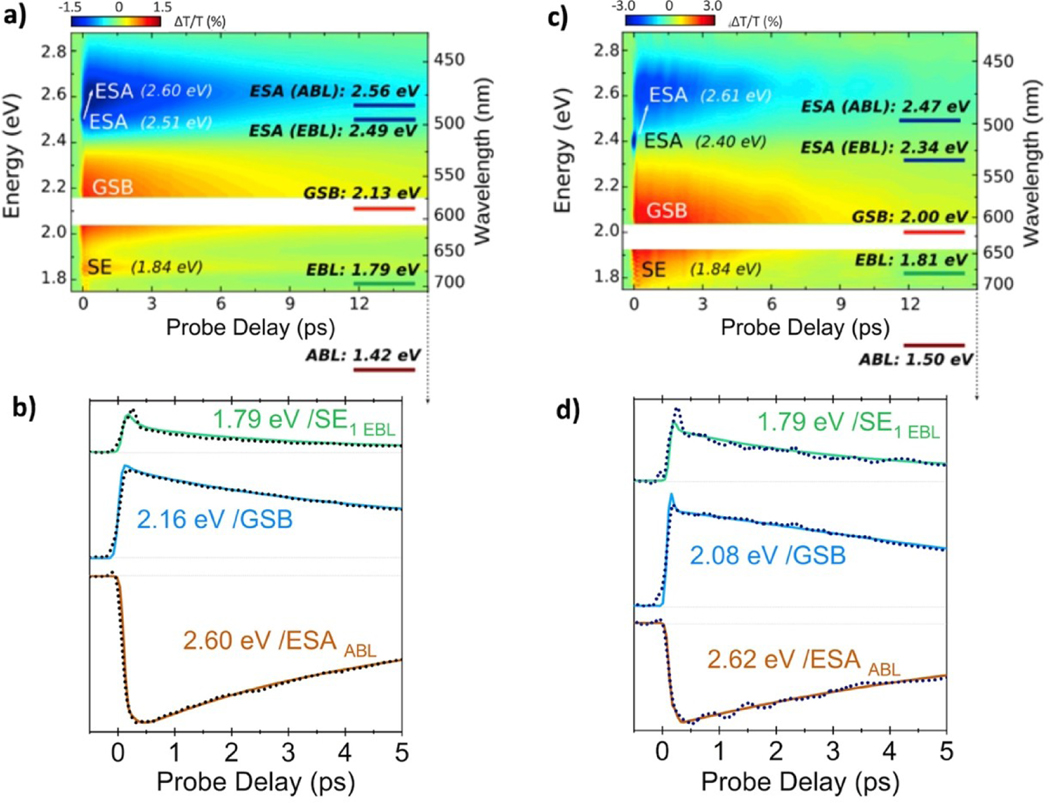
Ultrafast spectroscopy of M8 (a-b) and M10 (c-d), showing the experimental ΔT/T maps and average vertical excitation energies computed at the SS-CASPT2/ANO–S level (a,c) and four ΔT/T temporal traces at specific probe photon energies (b–d), with dotted lines corresponding to exponential fits.

Analogously to M4, we extended our combined experimental/theoretical analysis to the other two proteins, i.e. M8 and M10. The results are reported in Figure 5, which shows the experimental ΔT/T maps for both cases, their time-evolution at selected probe photon energies and the average energies computed at the SS-CASPT2 level of theory. In the ΔT/T map measured for the M8 protein (Figure 5a), two strong broad bands with opposite signs appear, peaked around 2.45–2.75 eV and 1.95–2.35 eV, assigned to ESA and GSB, respectively. In contrast to M4, the broad ESA band of M8 is found to be spectrally separated from the GSB and can be decomposed in two different signals: a first band centered around 2.50 eV, showing an ultrafast decay component (0.13 ps, close to the instrumental resolution, Figure 5b), and a second broader blue-shifted band, centered around 2.60 eV, which displays a slightly delayed build-up (see the temporal trace at 2.60 eV in Figure 5b). The comparatively narrow ESA peak observed at ≈ 2.51 eV is assigned to stimulated Raman scattering (SRS) from the aqueous solvent and corresponds to stimulated Raman loss at the intense 3400 cm ^1^ Raman peak of water. Once more, the experimental ESA band decomposition is supported by SS-CASPT2 computations, which predict an ESA signal from EBL at 2.49 eV (ESA_EBL_), being slightly blue-shifted with respect to ESA_ABL_ that lies at 2.56 eV, in quite good agreement with experiments. The time-resolved ESA signals (Figure 5b) and the global analysis in Figure S18 confirm that the ESA_ABL_ signal shows a delayed build-up (which corresponds to a positive band at ≈2.6 eV in the first DAS in Figure S18b), thus following the EBL→ABL bond relaxation pathway on the 100-fs timescale.

In the M8 ΔT/T spectrum, a second positive band (weaker that the GSB band) can be observed around 1.84 eV (Figure 5a), with a decay time of 2.65 ps. The origin of such band can be also clarified by our calculations. Indeed, we predict for M8 proteins two SE signals from EBL (SE_EBL_) and ABL (SE_ABL_) peaked at 1.79 and 1.42 eV, respectively. While the lower energy SE_ABL_ falls outside our experimental detection range, the positive ΔT/T time trace measured at 1.79 eV shows an ultrafast decay component (ca. 0.13 ps time constant, Figure 5b) that we can assign to decay of SE of the EBL state (SE_EBL_), consistently with the fast EBL→ABL relaxation decay mechanism expected for M8 (see Figure2). Moreover, our computations of the excited state manifold at the ABL geometries (see Figure S14 in the Supporting Information), reveal the existence of ESA_ABL_ signals lying in the spectral range between the GSB and SE_EBL_ signals (1.79–2.13 eV), suggesting that the ΔT/T map is spectrally congested in this region. In fact, the ‘pure’ GSB band is expected to be broader (1.80–2.35 eV) than how it appears in the map and its appearance is affected by the overlap with an ESA band in the 1.80–2.00 eV range, which tends to cancel it, and by the positive SE_EBL_ signal lying around 1.80 eV that allows emerging of its low-energy tail. The ultrafast spectroscopy of M10 (Figures 5c–d) is quite similar to that of the M8 protein. In this case, however, the full broad GSB band is clearly visible in the ΔT/T map, in contrast to M8.

Notably, our computations predict a smaller energy gap between the GSB and SE_EBL_ signals (1.81–2.00 eV) in M10 with respect to M8 (1.79–2.13 eV), explaining why the SE_EBL_ signal is concealed under the GSB band in the experimental map. Nevertheless, analysis of the signal decay at 1.79 eV confirms the presence of a fast component at the low energy tail, in agreement with the computed SE_EBL_ signal at 1.81 eV. The presence of a transition between EBL and ABL species is also confirmed by the analysis of the ESA band in M10. A strong instantaneous SRS peak at 2.4 eV, red shifted with respect to M8 due to the corresponding shift in pump pulse photon energy, is observed, while the ESA_ABL_ band centered at 2.61 eV has a clearly delayed rise (see Figure 5d) and features a 7.18 ps decay. Theory predicts a red shift of the ESA_ABL_ band by 0.13 eV with respect to the ESA_EBL_ one, so that experimental signatures of ESA_EBL_ are expected to be covered by the intense SRS peak. Finally, we note that the selected ΔT/T time traces in M10 (Figure 5d) are modulated by coherent oscillations (≈600 fs period,≈50 cm−^1^ frequency) generated by the ultrashort pump pulses. This mode is similar to the 60 cm−^1^ mode observed by Wang and co-workers in bovine rhodopsin ^[42]^ and attributed to a skeletal torsional motion of the chromophore and to the 80 cm ^1^ mode observed by Leonard and co-workers in artificial photoswitches.^[1]^ Such dynamical effects are not accounted for in the simulations reported here and would require further studies to assess the lineshape and dynamics of the pump-probe signals, as well as the state-hopping mechanisms at conical intersection seams. Still, the static approach adopted in this work allow us to assign the origin of the main signals in the experimental pump-probe spectra.

**Figure 6.**
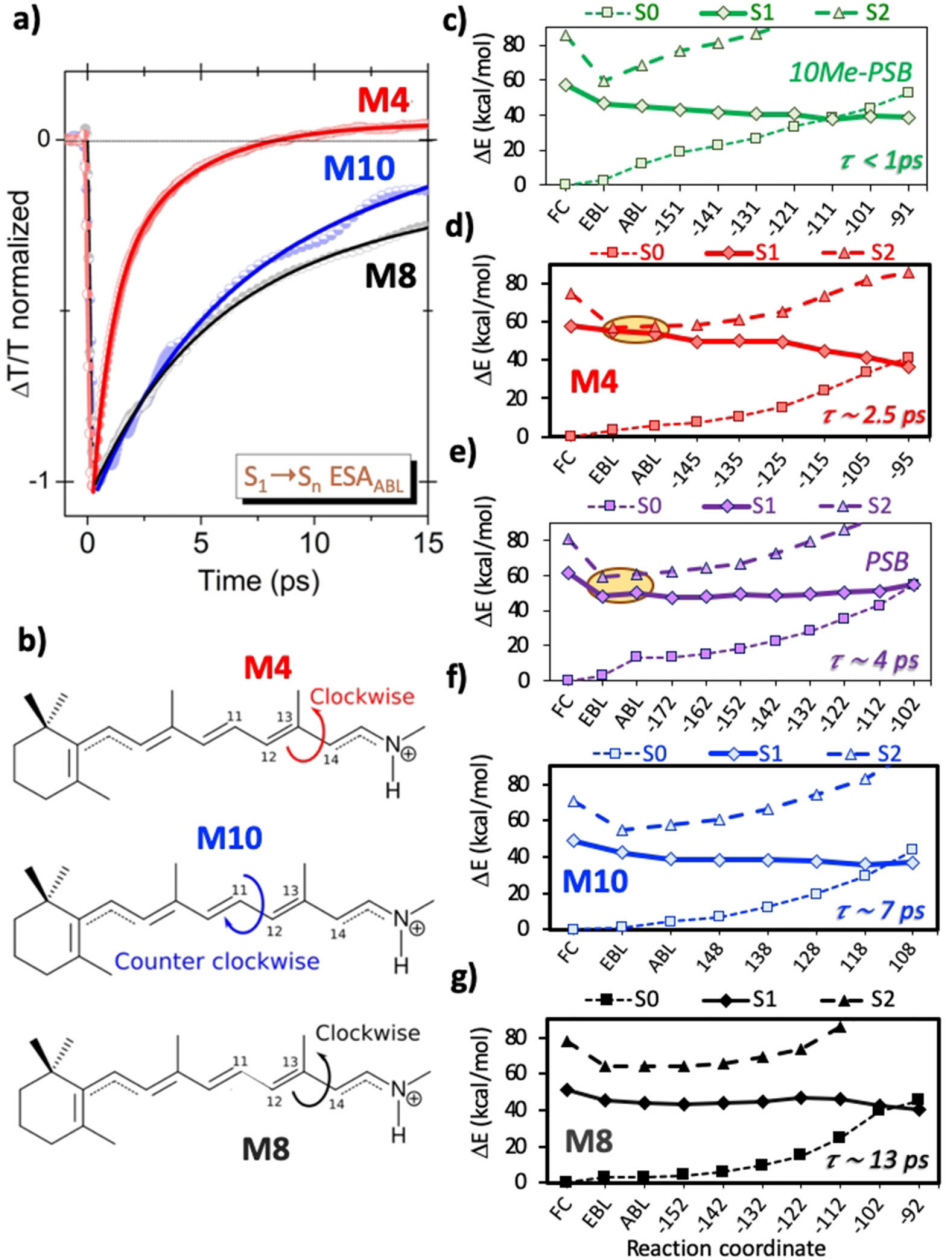
(a) Time evolution of the ESA_ABL_ signals in M4 (red), M8 (black) and M10 (blue) proteins, as representative of the S_1_ excited state decay. (b)Photoisomerization reaction directions around the C_11–_C_12_ and the C_13–_C_14_ bonds starting from the ABL structure. Photoisomerization MEPs in hCRBPII proteins and comparison with those of PSBs in solution, reproduced from ^[19]^. MEPs computed at the CASPT2 level are depicted for the clockwise rotation around the C_11–_C_12_ bond of 10Me-PSB (c, green lines) and PSB (e, magenta lines) in methanol, the clockwise rotation around the C_13–_C_14_ bond of M4 (d, red lines) and M8 (g, black lines), and the counter-clockwise rotation around the C_11–_C_12_ bond of M10 (f, blue lines).

The experimental pump-probe measurements provide evidence of a shorter excited-state lifetime for M4 with respect to the M8 and M10 proteins. This is clearly shown in Figure 6(a), monitoring the lifetime of the ESA_ABL_, which is the most representative signal of the overall S_1_ decay for all hCRBPII proteins. While the exploration of the C–C bond relaxation pathways and the characterization of SE and ESA transitions by multireference computations yielded a detailed and accurate interpretation of the observed ΔT/T signals, they do not provide direct interpretation of S_1_ lifetime trends. We have thus extended our computational study to the characterization of the PSB photoisomerization reaction MEP for each mimic, assuming it occurs from the bond-alternated (ABL) structure.

Considering as representative configurations of M4, M8 and M10 proteins those having the best agreement between vertical S_0_→S_1_ excitation energies and experimental absorption energies, constrained scans along the dihedral angle rotation around either the C_11–_C_12_ (as in rhodopsins) or the C_13–_C_14_ (as in bacteriorhodopsins) bond have been performed. The direction of the rotation (clockwise, CW, or counter-clockwise, CCW) has been chosen based on the pre-twisting resulting out of the ground state and excited-state geometries optimizations. In particular, pre-twisting in the CW direction around C_13–_C_14_ of M4 and M8 (see Figure S8 and S9 in the Supporting Information), and pre-twisting in the CCW direction around C_11_ C_12_ of M10 (see Figure S10 in the Supporting Information) have been found. If no pre-twist was observed, both possible directions have been considered for computing the photoisomerization MEPs (see Figure S16–S17 in the Supporting Information). The relevant dihedral angles have been modified with 10° increments, and the relaxation of the remaining degrees of freedom was performed at the CASSCF level (including three states in the state-averaged computations SA3-CASSCF), followed by CASPT2 energy corrections.

It is worth mentioning that, as shown in Figures S8-S13 in the Supporting Information, the geometrical deformations of the PSB in the ground or excited state optimized geometries of the hCRBPII proteins are not generally correlated to the S_1_/S_2_ energy gap. For instance, while a substantial heterogeneity of the dihedral angles defining the PSB planarity is found in the samples of M10 (see Figure S10) the corresponding S_1_/S_2_ energy gaps are found to be unvaried at the ABL S_1_ optimized geometries (Figure S13), as well as at EBL geometries. However, such geometrical deformations (C–C bonds elongation and deviations from planarity, i.e. pre-twistings) are particularly relevant for determining the most favorable photoisomerization pathways. In particular, the elongation of the C_13–_C_14_ bond mentioned above for M4, is generally accompanied by a significant distortion (in the CW direction) of the C_12–_C_13–_C_14–_C_15_ dihedral angle (from 0° in S_0_ to –25° in S_1_), see Figure S8, similarly (while CCW) to what occurs in bR.^[37]^ Analogously, for M8 all excited states optimized geometries feature non-planar C_12–_C_13–_C_14–_C_15_ dihedral angle, implying that both M4 and M8 will preferentially follow the CW C_13–_C_14_ photoisomerization pathway. On the other hand, PSBs in M10 already feature a CCW distorted C_10–_C_11–_C_12–_C_13_ dihedral angle in the ground-state optimized geometries, suggesting that the Q4R mutation has a significant impact on the photoisomerization pathways.

Figure 6(c–g) shows the computed MEPs for the three most relevant photoisomerization pathways of hCRBPII proteins involving CIs between the S_1_ and S_0_ surfaces (with all remaining computed pathways being reported in the Supporting Information) and the comparison with those of PSB and 10Me-PSB in methanol solution.^[19]^ The MEP for the CW C_13–_C_14_ photoisomerization of M4 shows how the S_1_ PES is decreasing steadily along the scan, crossing the S_0_ PES at a dihedral angle close to 105°. This PES topography is reminiscent of PSB photoisomerization with peaked CI funnels, such as that computed for the C_11–_C_12_ photoisomerization of 10Me-PSB^[19]^ reported in Figure 6c. However, the M4 protein features longer excited state lifetime (2.10 ps) with respect to the chemically modified solvated PSB (0.70 ps).^[19,36]^ This comparison clearly shows how the avoided crossing found during bond relaxation of M4 (see Figure 2a) and not observed in the 10Me-PSB,^[19]^ is the factor determining the difference in the lifetimes experimentally observed for M4 and 10Me-PSB. The CW C_11_-C_12_ photoisomerization of solvated “natural” PSB (see Figure 6e), instead, features an S_1_/S_2_ mixing as in M4, while its experimental lifetime is even longer than M4 (ca. 4 ps). The comparison of M4’s computed MEP with that of solvated PSB shows how, in this case, the difference in lifetimes could be explained in terms of the CI topography, since the PSB features an uphill PES along the photoisomerization and a corresponding sloped CI, in contrast with the downhill path and peaked CI of M4. The CCW C_11–_C_12_ photoisomerization of M10 (Figure 6f) is characterized by a rather flat PES and by the absence of an S_1_/S_2_ mixing, which somehow contrasts with the “long” experimental lifetime (ca. 7 ps). However, the small energy difference between GSB and SE signals found in the M10 pump-probe spectrum (ca. 0.20 eV, see Figure 5c) and the topography of the computed S_1_ photoisomerization MEP indicate that the tiny gain in potential energy along the relaxation from the FC region towards the CI seam could grant only a small kinetic energy to the excited state wavepacket traveling along the PES. This contrasts with the significant energy relaxation from FC to EBL found in the solvated PSB and can account for the difference in experimental S_1_ lifetime. In M8, the MEP of the CW C_13–_C_14_ photoisomerization features a sizable barrier of ca. 0.11 eV (i.e. 2.45 kcal/mol) (see Figure 6g), explaining why the lifetime for M8 (ca. 13 ps) is found to be longer than that of M10 (see Figure 6a). Thus, also the ground-state absorption energies can play a relevant role for controlling the excited decay, if the S_1_/S_2_ energy gap along the photoisomerization pathway and/or the CI topography are not the predominant factors. Finally, it is worth mentioning that, as shown in Figure S19 in the Supporting Information, in the hCRBPII pigments, independently on the spectral tuning, a space-saving bicycle pedal mechanism is observed along the computed MEP of PSBs’ photoisomerization.

## Conclusions

Our synergistic study combining ultrafast optical spectroscopy and theoretical simulations demonstrates how artificial protein design can achieve not only color tuning of the pigments but also fine control of the excited state properties of the retinal PSB chromophores. Point mutations of highly tunable hCRBPII host proteins, mimicking covalent retinal binding and photoisomerization in rhodopsin proteins, alter the environment of the organic chromophore determining the amount of intramolecular charge transfer in the spectroscopic state upon photo-excitation. This electrostatic effect in turn modifies the relative potential energy profiles of the covalent (S_2_) and ionic (S_1_) excited states along the C–C bond relaxation pathway, shaping the potential energy surface associated to the excited state decay.

Notably, this environmental effect has consequences on the potential energy profiles that are analogous to those observed upon chemical modification of the retinal chromophore, despite having quite different physical origin. However, these alterations (associated to the S_1_/S_2_ energy gap) alone do not explain the PSB’s excited state decays recorded experimentally in hCRBPII proteins, especially when they are compared to those of PSBs in solution. In fact, ultrafast transient absorption experiments demonstrate that modifications of the protein scaffold can effectively control not only the color of the biomimetic systems but also the excited state decay rates. We found remarkable agreement between theoretical predictions and experimental data, allowing assignment of the bands observed in the transient absorption signals and providing interpretation of the time-resolved data at the molecular level.

Two transient fluorescent intermediates, characterized by different bond length alternation patterns, are transiently populated during the early stage of excited state decay. These transient species, featuring spectroscopic fingerprints specific for each hCRBPII, have been resolved in this work and unambiguously assigned by first-principles simulations. The time evolution analysis of these spectroscopic signals yielded estimates of the PSB excited state lifetimes, which are significantly affected by the point mutations in the host protein. Theoretical (static) characterization of the photoisomerization pathways provided interpretation of these experimental evidences, showing that the measured excited state decay rates are associated to differences in the PESs along both the bond relaxation and the torsional modes, with the topography of the conical intersections with ground state playing also a relevant role.

Static characterization of photoisomerization MEPs indeed shed light on the complexity of the fine control of the PSB photophysics, which can be achieved only by simultaneously regulating three dominant components: i) the topography of the PES along the torsional motion that brings to the conical intersections, which generally represents the dominant factor but alone can be insufficient to predict PSB’s lifetime, since ii) the S_1_/S_2_ energy gap along the C–C bond relaxation coordinate can play a determinant role when the torsional MEP is similar between two systems (e.g. it explains why 10-methylated PSB in solution decays much faster than blue-shifted artificial pigments, like M4); and iii) the energy associated to the C–C bond relaxation from the Franck-Condon region can also bring a fundamental contribution when slow decays due to small S_1_/ S_2_ energy gap and/or flat PES are encountered along the torsional motion (as in solvated “natural” PSB and red-shifted pigments like M10).

We note that, while these three “static” factors have been individually recognized in literature, here their specific roles in regulating PSBs’ lifetime are finally determined by monitoring ultrafast transient absorption signals suitably interpreted by first-principles simulations. Further dynamic studies would be necessary to shed full light on the processes that finally determine the PSBs’ excited state decay. In summary, the combined study of excited state lifetimes and photoisomerization reaction pathways highlights the power of point mutations of the protein in tuning the photophysical/photochemical properties of the embedded organic chromophore, with effects comparable to those of chemical modifications and solvation. Taken together, our results grant fundamental knowledge for the design of biomimetic opto-electronic molecular devices.

## Supplementary Material

supinfo

## Figures and Tables

**Table 1.** Computed averaged vertical S_0_→S_1_ absorptions of the M4, M8 and M10 mimics in solution, compared to values of proteins in vacuum (crystal structures) and to experimental linear absorption maxima. Averaged permanent dipole moment difference, |Δμ|, between S_1_ and S_0_ states, and charge transfer (CT) characters of the transitions are also reported for solvated proteins.

	Level	QM/MM (eV) ^[a]^	Exp.	I A|x I (D)^[b]^	CT^[c]^

M4	CASPT2	2.67	2.4S	12.67	27%
	MS-PT2	2.47			
M8^[d]^	CASPT2	2.13	2.10	16.1S	47%
	MS-PT2	2.47			
M10	CASPT2	2.00	1.99	17.32	46%
	MS-PT2	2.13			

[a] Using ANO–S basis set.

[b] Computed at the CASSCF level.

[c] Based on CASSCF Mulliken charges and computed as percentage of (S_0_) positive charge moving from the C_12–_N to the C_5 –_C_11_ fragment in the S1 state.

[d] Considering the subset of M8’ configurations (see Supporting Information).
