# Supplementary material for: Control of Protonated Schiff Base Excited State Decay within Visual Protein Mimics: A Unified Model for Retinal Chromophores
Source: Chemistry. Author manuscript; Available in PMC 2022 Nov 25. (PMC8906800; doi:10.1002/chem.202102383)
Supplement: supinfo [file NIHMS1754295-supplement-supinfo.pdf]

Chemistry–A European Journal

Supporting Information

Control of Protonated Schiff Base Excited State Decay within Visual Protein Mimics: A Unified Model for Retinal Chromophores

Chemistry–A European Journal

Supporting Information

Control of Protonated Schiff Base Excited State Decay within Visual Protein Mimics: A Unified Model for Retinal Chromophores

Content:

Supplementary text
Figs. S1 to S19
Tables S1 to S13

Supplementary Text

Franck-Condon region

Ground state geometries. Considering the CRABPII proteins in the absence of explicit solvent molecules (apart from those water molecules present in the crystallographic structures) and optimizing the ground state geometries at the CASSCF level leads to computed vertical $S_0 \rightarrow S_1$ transition energies largely blue-shifted with respect to the experimental absorption maxima (see Table 1 in the main text), with blue-shifts of 0.88, 0.73 and 0.22 eV for M4, M8 and M10 respectively at the SS-CASPT2 level (and of 0.63, 0.64 and 0.41 eV at MS-CASPT2), while the expected accuracy of this QM/MM approach is around 0.2 eV. Changing the optimization method from CASSCF to DFT (B3LYP) or MP2 for the ground stat geometries did not correct this large blue-shift. Since the crystal structures (protonated using standard protonation states) are negatively charged, we have also considered proteins neutralized with Na^+ counterions, which still show analogous large blue-shifts. As showed in Figure S1, inspection of the X-ray structure of the hCRBPII proteins indicates that the PSB molecule (and in particular the ionone ring) is more exposed to solvent water molecules in the mimics than in natural opsin proteins, which are trans-membrane proteins that efficiently shield the PSB moiety from its external environment.

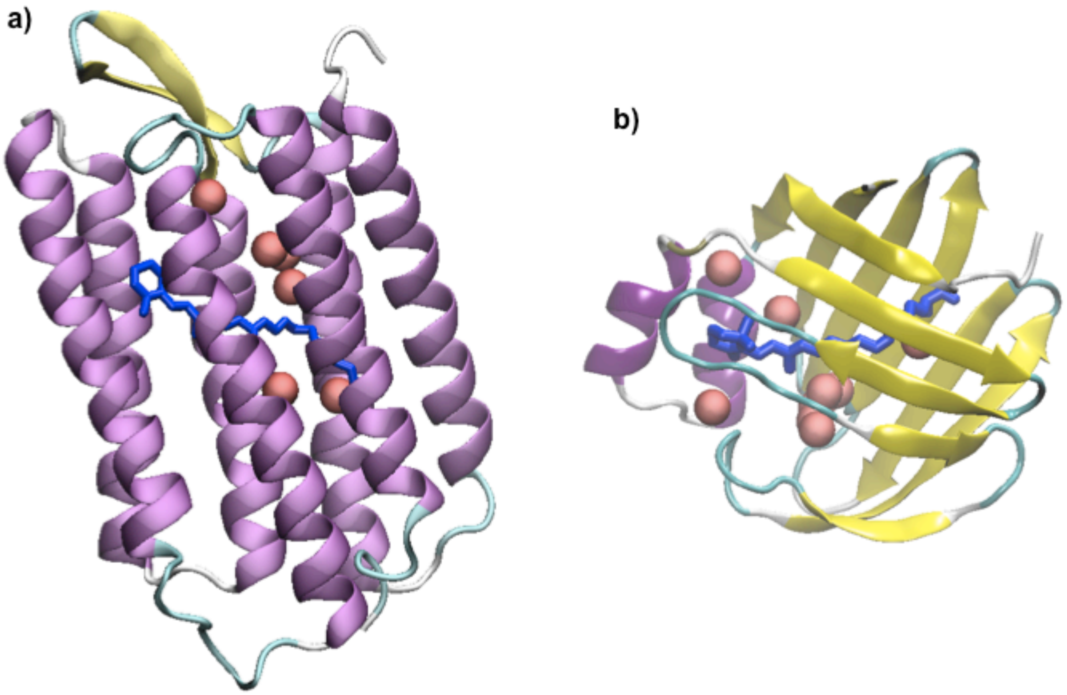

Figure S1. **Water accessibility of PSB binding site.** X-ray structures of the (a) bacteriorhodopsin (PDB code 1C3W) and (b) M8 hCRBPII mimics (PDB code 4EFG), showing the different exposure of the PSB (blue sticks) to solvent molecules, with crystal waters as red balls.

Thus, explicit solvent molecules are required for a realistic modeling of these systems. As described in the main text, we have followed the approach previously used for CRABPII proteins, performing classical molecular dynamics (MD) simulations and selecting a limited number (ten for each hCRBPII protein) of frames that have been subsequently refined with QM/MM optimizations to obtain the final ground state geometries.

The bond lengths and the dihedral values along the PSB polyene chain, obtained after CASSCF ground state optimizations at the QM/MM level, are collected for all the ten configurations of each protein. We present in this section the average values obtained. The bond lengths show very little deviation from configuration to configuration for a given protein, in contrast to dihedral angles (Figure S2). As can be seen, the PSB in M4 has a more pronounced bond length alternation in the C12-C15 region, and it is more distorted. As expected from the minor difference in the crystal structure, the PSBs in M8 and M10 have very similar geometry. Interestingly though, the ionone ring is more twisted in M8 than in M10, with dihedral angles values for the C5-C6-C7-C8 bond of 48.1° and 17.8° respectively, even if the only difference between M8 and M10 is the residue number 4 (Gln in M8, Arg in M10) located at the other side of the PSB.

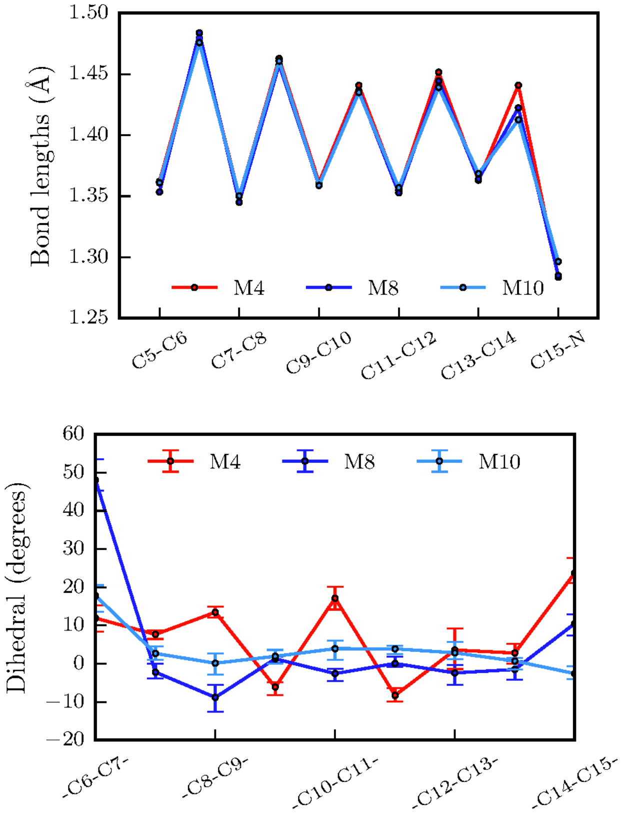

Figure S2. Average geometries (out of the ten configurations) after CASSCF optimizations. Top panel: bond length alternation in Å. Bottom panel : dihedral angles in degree, with error bars indicating the deviations obtained.

Excited states wavefunctions. In this section we analyze the wavefunctions obtained after CASSCF optimizations of the (ten) different configurations in the ground state. We also present the Perturbatively Modified CASSCF (PM-CASSCF) wavefunction, arising from the diagonalization of the effective Hamiltonian in the MS-CASPT2 approach.[1] All the transition energies are presented in eV, and are computed with the ANO-S basis set. For the wavefunction characters, only configurations with a weight of more that 0.09 were kept. « GS » refers to the closed shell determinant, « H » refers to Highest Occupied Molecular Orbital, « L » refers to Lowest Unoccupied Molecular Orbital, and the superscript « ^{2} » denotes a doubly excited transition. As indicated in the main text, the M8 wavefunctions feature two distinct behaviors among the ten solvent configurations selected from the MD sampling. Indeed, in four cases (Table S3), the S_1 and S_2 states are well separated in energy, with an S_1 state dominated by the $H \rightarrow L$ configuration and an S_2 state dominated by the $(H \rightarrow L)^2$ configuration (as for M10), while in all the others, the same mixing as for M4 appears. This translates into two sets of vertical absorptions, where the 'M4-like' (namely M8'') configurations give highly blue-shifted values, while the 'M10-like' (namely M8') configurations provide a better agreement with the experimental linear absorption spectrum. Given the proximity of M8 and M10, we expect an overall similar behavior for these two systems. However, we obtained more M8'' configurations than M8', indicating that M8 is a challenging case for our (limited) sampling approach, possibly requiring quite extended sampling of solvent configurations and accurate refinement of the initial crystal structure. Obtaining a large and very accurate sampling of the

conformational space of solvated hCRBPII proteins is out of the scope of this work, where the MD sampling has been performed to alleviate the limitations of computing transition energies on top of crystal structures in gas-phase and to obtain at least few representative protein structures in order to investigate the decay dynamics of the photogenerated S_1 state in the PSB.

Table S1. CASSCF wavefunctions, CASPT2 transition energies (ΔE in eV) and oscillator strengths (*f*) obtained for M4 protein.

Root	Configuration weights	$\Delta E(S_0 \rightarrow S_n)$, eV	*f*
1	GS 0.75		
	H → L 0.34, $(H \rightarrow L)^2$ 0.13, H → L+1 0.13	2,54	0,45
	H → L 0.35, $(H \rightarrow L)^2$ 0.15	3,07	0,63
2	GS 0.75		
	H → L 0.31, $(H \rightarrow L)^2$ 0.14, H → L+1 0.14	2,59	0,41
	H → L 0.38, $(H \rightarrow L)^2$ 0.14	3,03	0,64
3	GS 0.75		
	H → L 0.42, H → L+1 0.12	2,38	0,58
	H → L 0.27, $(H \rightarrow L)^2$ 0.19	3,09	0,52
4	GS 0.75		
	H → L 0.34, H → L+1 0.13, $(H \rightarrow L)^2$ 0.13	2,55	0,45
	H → L 0.35, $(H \rightarrow L)^2$ 0.15	3,08	0,61
5	GS 0.75		
	H → L 0.51, H → L+1 0.11	2,27	0,67
	$(H \rightarrow L)^2$ 0.23, H → L 0.19, H-1 → L 0.11	3,19	0,39
6	GS 0.75		
	H → L 0.27, $(H \rightarrow L)^2$ 0.16, H → L+1 0.13	2,67	0,38
	H → L 0.43, $(H \rightarrow L)^2$ 0.12	2,97	0,75
7	GS 0.75		
	H → L 0.50, H → L+1 0.11	2,33	0,68
	$(H \rightarrow L)^2$ 0.22, H → L 0.19, H-1 → L 0.11	3,21	0,4
8	GS 0.75		
	H → L 0.56	2,85	0,86
	$(H \rightarrow L)^2$ 0.22, H → L 0.13, H → L+2 0.13	2,92	0,19
9	GS 0.75		
	H → L 0.44, $(H \rightarrow L)^2$ 0.12	2,42	0,61
	H → L 0.24, $(H \rightarrow L)^2$ 0.19	3,13	0,48
10	GS 0.75		
	H → L 0.52, H → L+1 0.11	2,26	0,66
	$(H \rightarrow L)^2$ 0.23, H → L 0.17, H-1 → L 0.12	3,21	0,36

Table S2. PM-CASSCF wavefunctions, MS-CASPT2 transition energies (ΔE in eV) and oscillator strengths (*f*) obtained for M4 protein.

Root	Configuration weights	$\Delta E(S_0 \rightarrow S_n)$, eV	*f*
1	GS 0.77		
	H → L 0.66	2,44	1,62
	$(H \rightarrow L)^2$ 0.28, H-1 → L 0.14	3,95	0,086
2	GS 0.77		
	H → L 0.65	2,44	1,55
	$(H \rightarrow L)^2$ 0.28, H-1 → L 0.14	3,95	0,093
3	GS 0.77		
	H → L 0.67	2,43	1,74
	$(H \rightarrow L)^2$ 0.29, H-1 → L 0.15	3,82	0,04
4	GS 0.77		
	H → L 0.65	2,45	1,61
	$(H \rightarrow L)^2$ 0.28, H-1 → L 0.14	3,96	0,077
5	GS 0.79		
	H → L 0.68	2,45	1,81
	$(H \rightarrow L)^2$ 0.30, H-1 → L 0.15	3,76	0,022
6	GS 0.77		
	H → L 0.64	2,52	1,65
	$(H \rightarrow L)^2$ 0.28, H-1 → L 0.14	3,98	0,13
7	GS 0.78		
	H → L 0.68	2,49	1,79
	$(H \rightarrow L)^2$ 0.29, H-1 → L 0.15	3,78	0,019
8	GS 0.76		
	H → L 0.61	2,58	1,48
	$(H \rightarrow L)^2$ 0.26, H-1 → L 0.12	3,96	0,27
9	GS 0.77		
	H → L 0.67	2,46	1,68
	$(H \rightarrow L)^2$ 0.29, H-1 → L 0.15	3,78	0,029
10	GS 0.78		
	H → L 0.67	2,43	1,75
	$(H \rightarrow L)^2$ 0.29, H-1 → L 0.15	3,70	0,026

Table S3. CASSCF wavefunctions, CASPT2 transition energies (ΔE in eV) and oscillator strengths (*f*) obtained for M8 protein. Bold values refer to the M8' configurations, while the rest is defined as M8''.

Root	Configuration weights	$\Delta E(S_0 \rightarrow S_n)$, eV	*f*
1	GS 0.76		
	H → L 0.57	2,34	0,81
	(H → L)2 0.26, H → L 0.13, H-1 → L 0.13	3,39	0,32
2	GS 0.76		
	H → L 0.57	2,32	0,79
	$(H \rightarrow L)^2$ 0.26, H → L 0.13, H-1 → L 0.13	3,40	0,33
3	GS 0.76		
	**H → L 0.66**	**2,05**	**1,03**
	$(H \rightarrow L)^2$ 0.31, H-1 → L 0.19	3,25	0,15
4	GS 0.76		
	**H → L 0.63**	**2,16**	**0,92**
	$(H \rightarrow L)^2$ 0.30, H-1 → L 0.16	3,37	0,2
5	GS 0.76		
	H → L 0.51	2,43	0,72
	$(H \rightarrow L)^2$ 0.24, H → L 0.20, H-1 → L 0.11	3,36	0,43
6	GS 0.76		
	**H → L 0.64**	**2,25**	**0,96**
	$(H \rightarrow L)^2$ 0.29, H-1 → L 0.17	3,40	0,18
7	GS 0.76		
	H → L 0.52	2,49	0,74
	$(H \rightarrow L)^2$ 0.24, H → L 0.19, H-1 → L 0.12	3,42	0,41
8	GS 0.76		
	H → L 0.50	2,44	0,7
	$(H \rightarrow L)^2$ 0.24, H → L 0.20, H-1 → L 0.11	3,39	0,44
9	GS 0.76		
	**H → L 0.64**	**2,19**	**0,98**
	$(H \rightarrow L)^2$ 0.30, H-1 → L 0.17	3,35	0,17
10	GS 0.76		
	H → L 0.58	2,36	0,82
	$(H \rightarrow L)^2$ 0.27, H-1 → L 0.13, H → L 0.13	3,43	0,31

Table S4. PM-CASSCF wavefunctions, MS-CASPT2 transition energies (ΔE in eV) and oscillator strengths (*f*) obtained for M8 protein.

Root	Configuration weights	$\Delta E(S_0 \rightarrow S_n)$, eV	*f*
1	GS 0.79		
	H → L 0.70	2,55	1,83
	$(H \rightarrow L)^2$ 0.32, H-1 → L 0.17	3,80	0,03
2	GS 0.79		
	H → L 0.70	2,55	1,87
	$(H \rightarrow L)^2$ 0.32, H-1 → L 0.17	3,83	0,03
3	GS 0.79		
	H → L 0.68	2,40	1,96
	$(H \rightarrow L)^2$ 0.32, H-1 → L 0.20	3,45	0,10
4	GS 0.79		
	H → L 0.69	2,50	1,94
	$(H \rightarrow L)^2$ 0.32, H-1 → L 0.19	3,63	0,06
5	GS 0.79		
	H → L 0.70	2,55	1,79
	$(H \rightarrow L)^2$ 0.31, H-1 → L 0.16	3,93	0,03
6	GS 0.79		
	H → L 0.70	2,55	1,85
	$(H \rightarrow L)^2$ 0.32, H-1 → L 0.19	3,63	0,05
7	GS 0.79		
	H → L 0.70	2,59	1,73
	$(H \rightarrow L)^2$ 0.31, H-1 → L 0.17	3,95	0,03
8	GS 0.79		
	H → L 0.70	2,56	1,82
	$(H \rightarrow L)^2$ 0.32, H-1 → L 0.17	4,01	0,03
9	GS 0.79		
	H → L 0.70	2,53	1,92
	$(H \rightarrow L)^2$ 0.32, H-1 → L 0.19	3,59	0,05
10	GS 0.79		
	H → L 0.70	2,58	1,84
	$(H \rightarrow L)^2$ 0.32, H-1 → L 0.17	3,84	0,02

Table S5. CASSCF wavefunctions, CASPT2 transition energies (ΔE in eV) and oscillator strengths (*f*) obtained for M10 protein.

Root	Configuration weights	$\Delta E(S_0 \rightarrow S_n)$, eV	*f*
1	GS 0.79		
	H → L 0.65	1,97	1,28
	$(H \rightarrow L)^2$ 0.27, H-1 → L 0.27	2,96	0,20
2	Gs 0.78		
	H → L 0.63	2,01	1,20
	$(H \rightarrow L)^2$ 0.27, H-1 → L 0.25	3,00	0,21
3	GS 0.77		
	H → L 0.62	2,02	1,19
	$(H \rightarrow L)^2$ 0.28, H-1 → L 0.24	3,03	0,20
4	GS 0.78		
	H → L 0.63	1,97	1,17
	$(H \rightarrow L)^2$ 0.26, H-1 → L 0.26	3,00	0,22
5	GS 0.77		
	H → L 0.62	2,02	1,19
	$(H \rightarrow L)^2$ 0.28, H-1 → L 0.24	3,03	0,20
6	GS 0.77		
	H → L 0.62	2,04	1,15
	$(H \rightarrow L)^2$ 0.27, H-1 → L 0.25	3,05	0,22
7	GS 0.77		
	H → L 0.62	2,01	1,18
	$(H \rightarrow L)^2$ 0.27, H-1 → L 0.25	3,01	0,21
8	GS 0.79		
	H → L 0.65	1,93	1,30
	$(H \rightarrow L)^2$ 0.28, H-1 → L 0.27	2,90	0,20
9	GS 0.78		
	H → L 0.65	1,99	1,24
	$(H \rightarrow L)^2$ 0.27, H-1 → L 0.27	2,99	0,21
10	GS 0.75		
	H → L 0.58	2,10	1,02
	$(H \rightarrow L)^2$ 0.26, H-1 → L 0.22	3,13	0,24

Table S6. PM-CASSCF wavefunctions, MS-CASPT2 transition energies (ΔE in eV) and oscillator strengths (*f*) obtained for M10 protein.

Root	Configuration weights	$\Delta E(S_0 \rightarrow S_n)$, eV	*f*
1	GS 0.80		
	H → L 0.64	2,07	1,70
	$(H \rightarrow L)^2$ 0.27, H-1 → L 0.26	3,01	0,27
2	GS 0.80		
	H → L 0.64	2,14	1,75
	$(H \rightarrow L)^2$ 0.28, H-1 → L 0.25	3,07	0,24
3	GS 0.80		
	H → L 0.64	2,17	1,78
	$(H \rightarrow L)^2$ 0.28, H-1 → L 0.24	3,10	0,22
4	GS 0.80		
	H → L 0.63	2,10	1,73
	$(H \rightarrow L)^2$ 0.27, H-1 → L 0.26	3,07	0,28
5	GS 0.80		
	H → L 0.64	2,16	1,75
	$(H \rightarrow L)^2$ 0.28, H-1 → L 0.24	3,10	0,22
6	GS 0.80		
	H → L 0.63	2,18	1,73
	$(H \rightarrow L)^2$ 0.27, H-1 → L 0.25	3,12	0,24
7	GS 0.80		
	H → L 0.64	2,16	1,76
	$(H \rightarrow L)^2$ 0.27, H-1 → L 0.25	3,09	0,24
8	GS 0.80		
	H → L 0.64	2,03	1,74
	$(H \rightarrow L)^2$ 0.27, H-1 → L 0.26	2,96	0,28
9	GS 0.80		
	H → L 0.64	2,09	1,67
	$(H \rightarrow L)^2$ 0.27, H-1 → L 0.26	3,04	0,28
10	GS 0.80		
	H → L 0.64	2,29	1,82
	$(H \rightarrow L)^2$ 0.28, H-1 → L 0.22	3,26	0,17

Permanent dipole moments. The permanent dipoles, extracted from the CASSCF wavefunctions, have been collected for all the ten configurations for each protein, and are displayed in Figure S3. In M4, large variations in direction are observed for the S_1 dipoles, which in some cases overlap with S_2 dipoles. The direction of the dipole moment in S_0 is consistent with the localization of the charge in the ground state on the nitrogen side of the PSB molecule. Upon excitation, a charge transfer along the PSB conjugated chain is expected. However, the direction of the S_1 dipole moments indicates a quite ineffective charge transfer in this protein. The intensity of the dipole moment also decreases sharply when going from S_0 to S_1, which indicates that the charge separation is low on the excited state. Finally, here again the huge influence of the HBN is clearly noticeable, given the wide variations in directions.

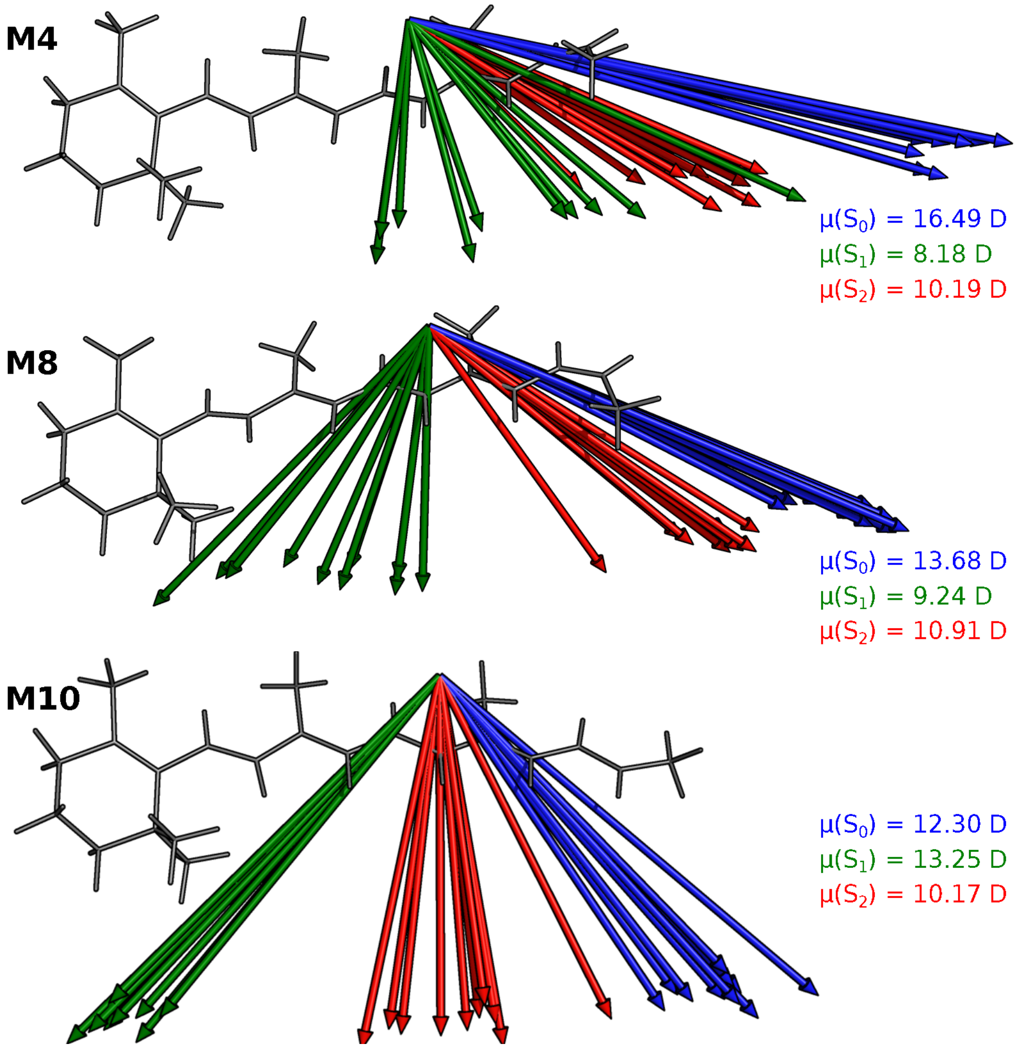

Figure S3. Distribution of the permanent dipole moments obtained for the three proteins, for the states S_0 (blue), S_1 (green) and S_2 (red), computed at the CASSCF/ANO-S level.

In M8, there is also a wide variation in the directions in S_1 dipole moments. However, the S_1 dipole moments are more oriented toward the ionone ring than in M4, indicating a more effective charge transfer in this protein. The increasing value of the intensity of this dipole is another proof of this more efficient charge transfer. Finally, we can notice that the S_0 dipoles are less intense than in M4, which indicate a lower localization of charges in the ground state.

Finally, in M10, the dispersion of directions is reduced, consistently with the efficient shielding of the PSB from external environment. Also, the intensities of S_1 and S_0 dipoles are in the same range (13.25 D and 12.30 D respectively), indicating a very effective charge transfer.

The evolution of the norm of permanent dipole moment vector difference $\Delta\mu(S_0 \rightarrow S_1)$, i.e. $\mu(S_0)–\mu(S_1)$, from M4 to M10, as depicted in Figure S4, further indicates that the charge transfer intensity is increasing going from M4 to M10. In the three proteins, the direction of the dipole moment differences shows the transfer of positive charge from the nitrogen side to the ionone side of the PSB. The values of $\Delta\mu(S_0 \rightarrow S_1)$ in M8' and M10 are similar.

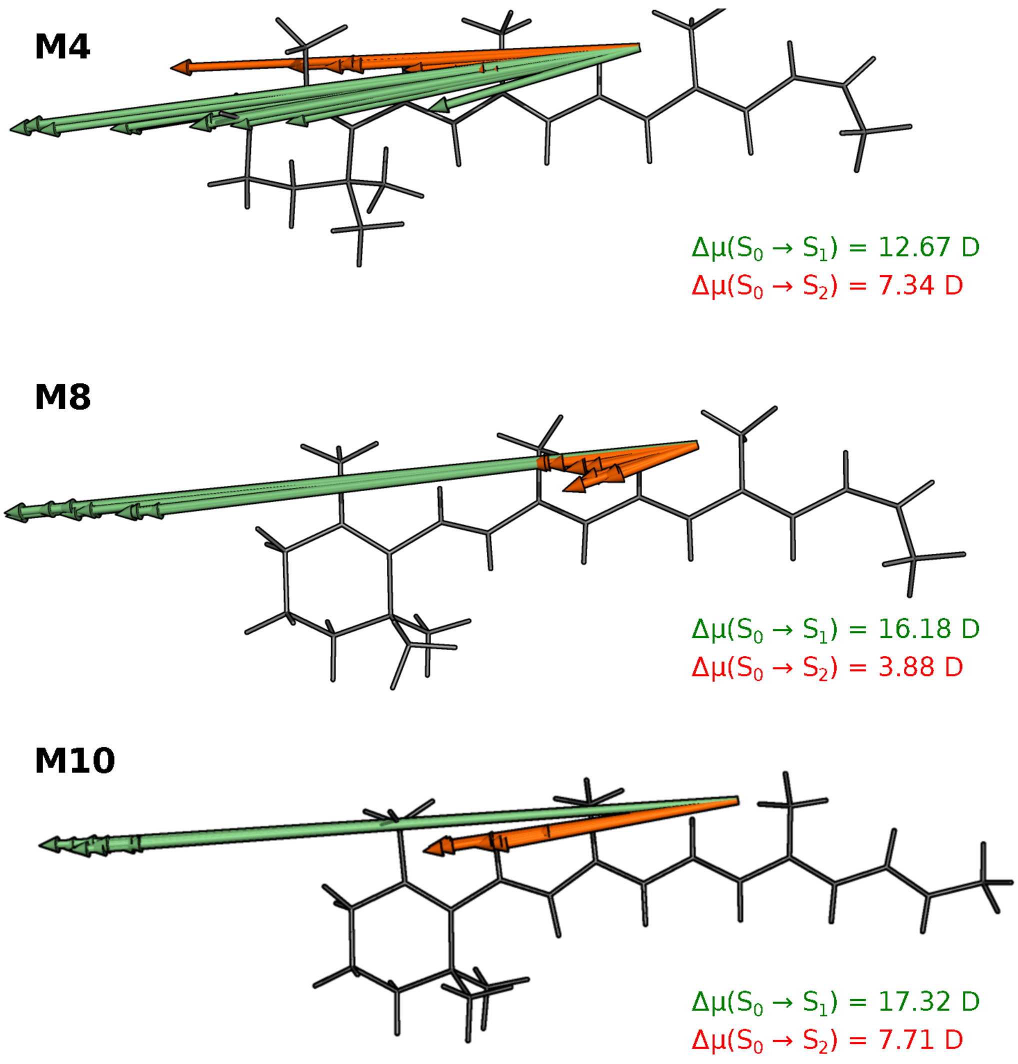

Figure S4. Distribution of the permanent dipole moment vector difference, with $\Delta\mu(S_0 \rightarrow S_1)$ in green and $\Delta\mu(S_0 \rightarrow S_2)$ in orange, obtained for the three proteins at the CASSCF/ANO-S level.

Partial charges analysis. The Mulliken charge analysis is carried out on the CASSCF wavefunctions. For each solvent configuration, the charges are computed for all atoms while the hydrogen charges are summed up on heavy atoms. The PSB molecule is divided into two fragments by cutting it at the C11–C12 bond. The two fragments will be referred to as the lysine and ionone part, respectively. The charges of each atom in each fragment are summed up, and the average over the ten snapshots of each protein is computed. The results are presented in Figure S5. The stronger localization of the charge on the lysine side in M4 in the ground state (S0) compared to M8 and M10 is clearly observed. The lysine side indeed accounts for around 90% of the positive charge in M4, whereas this amounts to around 75% in M10. The charge transfer efficiency is computed as the amount of charge that is transferred from one side to the other upon excitation. We found that on average, 27%, 41% and 46% of the charge initially on the lysine side is transferred to the ionone side for M4, M8 and M10 respectively. For completeness, by taking into account the M8' set of M8 configurations, the amount of charge transfer from the lysine side to the ionone side rises to 47%, and it is therefore very close to M10. These results confirm our conclusion that the charge transfer is significantly reduced in M4 with respect to what happens in the two other systems. As mentioned in the main text, PSB in M4 behaves like in methanol solution, as the computed internal charge transfer is comparable to what has previously been found for solvated PSB.

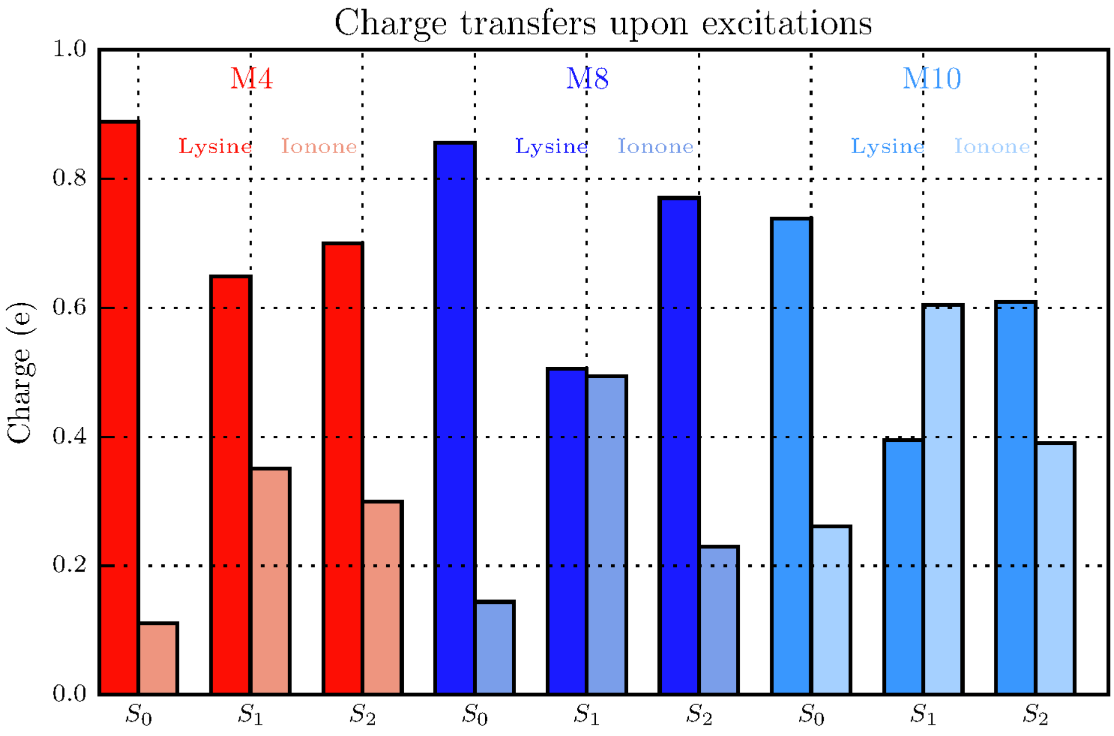

Figure S5. Averaged Mulliken charges summed up on the Lysine part (from N to C12) and on the Ionone part (from C11 to ionone ring).

To investigate more in depth on the repartition of the charge in the different systems, a per-atom analysis was carried out, in which we consider only the atoms in the conjugated chain of the PSB.

In this analysis, the charges carried by methyl groups are summed on the backbone heavy atoms the methyl belongs to. For instance, all the charges in the ionone ring are added to C5. The charges obtained are then averaged for each state in each protein, considering only M8' configurations for M8. The graph obtained is presented in Figure S6. The C12-C13 and C13-C14 bonds in the S_0 state of M4 and M8 proteins appear more polarized than in M10 proteins, which notably relates to the reactivity of the C13-C14 bond in the photoisomerization pathways showed in Figures S16-S17. In M10, the positive charge is evenly spread across the C13 – C8 region. The positive charge also appears strongly located on the N – C15 bond in both M4 and M8. When exciting M4 to S_1 , the charge of C10 and C8 increases to positive values, while the polarization of bonds in the C13 – C10 region is inverted. Overall, the polarization of C-C bonds is reduced in S_1. In M8, similar situation is observed with respect to M4, although a far more positive charge transfer is evident from the charge increase in the C10 – C7 region. In M10, a different picture appears. As expected from the bar diagrams presented below (Figure S5), the positive charge is transferred toward the ionone ring upo photoexcitation.

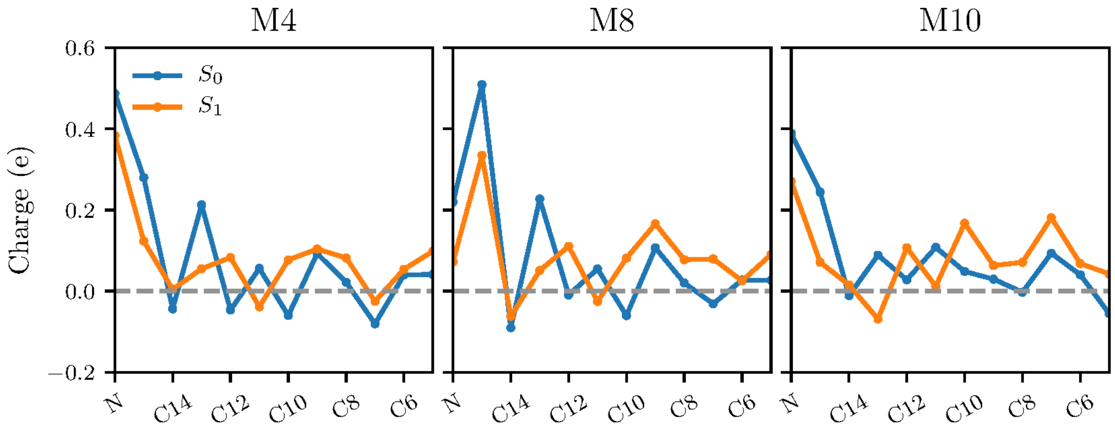

Figure S6. Averaged Mulliken charges (CASSCF/ANO-S) along the polyene chain of the PSB moiety in the three proteins. The charges of hydrogens and methyl groups have been summed up on the heavy atoms of the chain. Note that for M8 only the M8' configurations are considered.

To investigate more in depth on the effect of the environment on the spectroscopic properties of the embedded PSBs, we extracted the PSB molecule as defined in the high layer from the proteins, after QM/MM geometry optimizations. Without any reoptimization, we re-computed the energies of the S_0, S_1 and S_2 states at the SS- and MS-CASPT2 levels. By calculating the difference between the energies obtained in protein and those obtained in vacuum, we expect to get insight on differential effects of the set of point charges used for describing the protein on the different states involved. All the results in this section were computed using the 6-31G∗ basis set, and are reported in Table S7.

Table S7. Averaged vertical excitation energies and corresponding standard deviations computed for M4, M8 and M10 in protein and in vacuum (i.e. by removing all electrostatic contributions). All energies are in eV.

Protein	Level	Average		Std. Deviation	
		In prot.	In vacuo	In prot.	In vacuo
M4	CASPT2	2.66	1.98	0.18	0.01
	MS-CASPT2	2.58	2.24	0.04	0.02
M8	CASPT2	2.47	2.17	0.14	0.02
	MS-CASPT2	2.63	2.40	0.07	0.02
M10	CASPT2	2.09	2.00	0.05	0.01
	MS-CASPT2	2.24	2.22	0.07	0.01

The excitation energies obtained for the PSBs extracted from the three proteins (upon QM/MM optimizations) are close to one another (1.98 eV, 2.17 eV and 2.00 eV for M4, M8 and M10 respectively at SS-CASPT2 level), indicating that the tuning of the vertical absorption observed experimentally is mainly due to the environment (the solvated protein scaffold), and that the geometry of the PSB plays a minor role. Standard deviations of the excitation energies obtained in vacuum for the ten selected configurations (for each protein) are very low, and indicate that the PSB geometry and the vertical absorptions are very stable along each trajectory. The resulting values in vacuum are in good agreement with previous computational studies in gas-phase, and the blue-shift of M8 can be attributed to the twisted conformation of the ionone ring obtained (see Figure S2). It is worth noting that for M10 the results in protein and in vacuum are quite similar, pointing out how the PSB is efficiently shielded from the external environment in the M10 protein, and it behaves very closely to a PSB molecule in gas phase. Here, we evaluate the electrostatic effect of the protein scaffold as the energy difference

$$E_{el} = E_{QM(QM/MM)} - E_{QM(vacuum)}$$

where $E_{QM(QM/MM)}$ is the QM energy obtained for the PSB embedded in the solvated protein scaffold, and $E_{QM(vacuum)}$is the QM energy of the PSB in the gas phase, considered in the exact same geometry. This difference is computed for the S_0, S_1 and S_2 states for all the snapshots considered before. A negative difference indicates that the protein stabilizes the state under consideration. The results (computed with CASPT2 QM energies) obtained for the three proteins are presented in Figure S7. First, one can notice that the solvated protein scaffold stabilizes all the states in all proteins, due to the overall electrostatic stabilization of the charged PSB species by the environment. In particular, the positive charge carried out by the PSB PSB is stabilized by the overall negative charges of the M4, M8 and M10 proteins, carrying charges of -3, -5, -4, respectively (as determined by standard protonation states), with total systems neutralized by Na ions in solution. However, the overall stabilization in M4 and M8 is higher than in M10. This is most likely due to the mutation of Gln to Arg at position 4 in M10, leading to another positive charge at close proximity with the protonated Schiff base, thus less stabilizing the system. However, the states are not all equally affected. In M4, the solvated protein stabilizes more the covalent S_0 and S_2 states than the ionic S_1 state, leading to a blue shift of the vertical absorption with respect to gas-phase. The PSB positive charge is mainly located on the nitrogen in the ground state and it is expected to be stabilized by the HBN involving the nearby water molecule and Gln4 residue in M4 and M8, thus stabilizing S_0 and S_2 more than in M10. In M10, in fact, all the states are similarly stabilized, and no specific electrostatic effect can be observed. This is consistent with the effective shielding from external environment in M10. Focusing on the M8' configurations of M8 (i.e. configurations 03, 04, 06 and 09), a similar feature is observed, in which no differential effect is found for the three states.

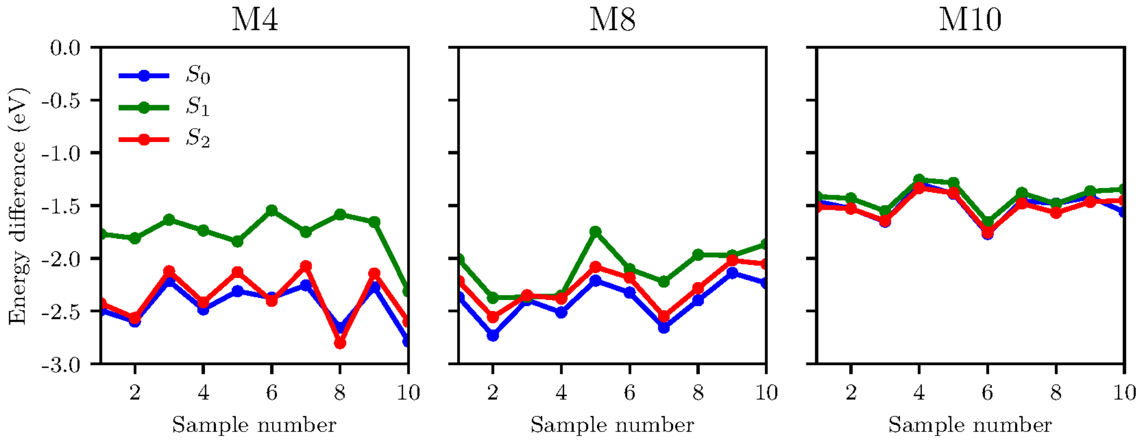

Figure S7. Averaged electrostatic effect of the protein scaffold on the ground and first two excited states of the PSB, in the three proteins (CASPT2/ANO-S energies).

Photophysical and photochemical properties

Excited state geometries. Optimizing the excited state in hCRBPII proteins is challenging, mainly due to root flipping that can occur. This has especially been the case for all the configurations in M4, and for some configurations in M8. At the CASSCF level, the H → L root is found to be root number 3 for all configurations in M4. This was also the case for the M8'' configurations of M8. In M10 and the M8' configurations of M8, the H → L root is always root 2. The case of M4, as highlighted in the main text, is highly similar to the one of PSB in solution reported previously. Indeed, the PSB optimizations give always an EBL-like geometry, characterized by even bond lengths in the conjugated chain of the PSB. However, some variability of the geometrical parameters among the various configurations is observed, as can be seen in Figure S8. Notably, most configurations have a strong elongation of the C13–C14 bond, from an average of 1.363 Å in the ground state to 1.447 Å in the excited state, although it varies from 1.437 Å to 1.459 Å.

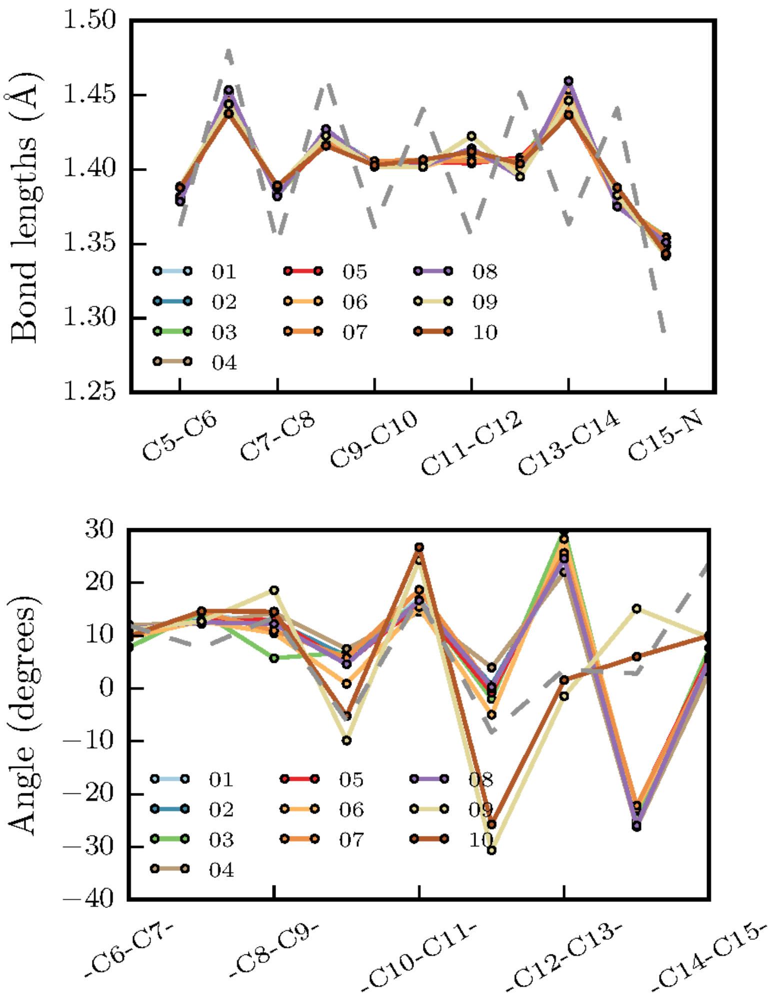

Figure S8. Geometrical parameters obtained after excited state optimizations in M4. Dashed gray lines indicate ground state average geometry. Top: bond lengths; Bottom: deviations from planarity of dihedral angles along the PSB conjugated chain.

The deviations from planarity of the PSB conjugated chain show that two configurations stand out. Indeed, most structures show strong deviations around the C12–C13 and C13–C14 bonds, whereas not much change occurs in this region for configurations 09 and 10. These two have strong distortions around the C11–C12 bond instead. This change of behavior stresses once again the importance of the initial sampling. Indeed, the HBN conformation around the PSB seems to strongly influence the excited state potential energy surface, by favoring some distortions over others.

For M8, the M8'' set leads to EBL-like optimized geometries, while the optimizations of the configurations belonging to the M8' set (except configuration 06) give ABL-like geometries. It is worth to notice that configuration 06 lies in between these two situations, with a BLA pattern reminding the one labeled ABL* in a recent study on solvated PSB molecules. The most elongated bond in the ABL optimized configurations is the C13 – C14 bond.

All configurations show a twisting around the C13–C14 bond, and this does not depend on the BLA pattern, although the ABL configurations have a stronger twisting. Another feature of the excited state geometries is that the ionone ring gets planarized, which is in line with the double bond character acquired by C6–C7 bond upon excitation (from 1.484 Å in the ground state to 1.425 Å in the excited state).

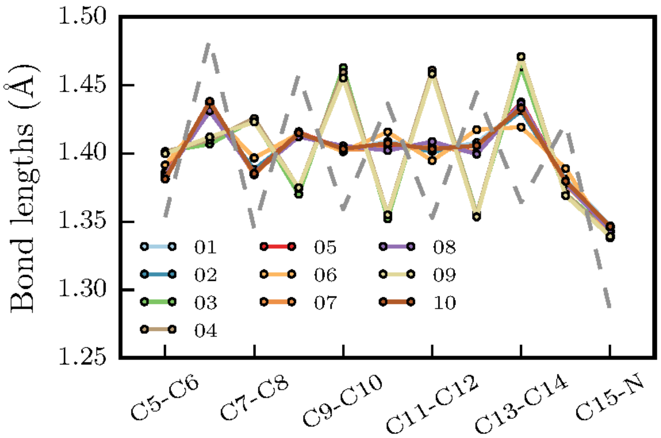

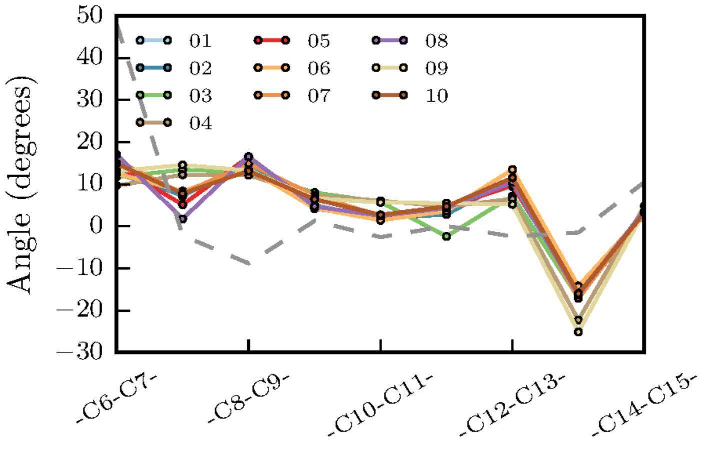

Figure S9. Geometrical parameters obtained after excited state optimizations in M8. Dashed gray lines indicate ground state average geometry. Top: bond lengths; Bottom: deviations from planarity of dihedral angles along the PSB conjugated chain.

After optimization of the H → L root in the M10 protein, the geometries obtained are ABL, featuring inversion of bond lengths of the conjugated chain of the PSB molecule, as obtained for 10-Methylated PSB in the solvated PSBs. The bond lengths obtained, as well as the deviations from planarity of the dihedrals are presented in Figure S10. Differently from M4 and M8, all the bond lengths obtained for M10 geometries are highly similar among them. The highest elongation is obtained for the central C11–C12 bond, which goes from an average of 1.357 Å in the ground state to 1.466 Å in the excited state. Concerning the dihedral angles, although some variety is observed, all the configurations share the same global parameters. Overall, they are fairly close to the averaged ground state structure. However, the twisting around the C11–C12 bond has significant variation along the trajectory, some configuration (like configuration 07) featuring a very strong distortion (up to 30°), while some others are nearly planar all along the chain (such as configuration 08 and configuration 04).

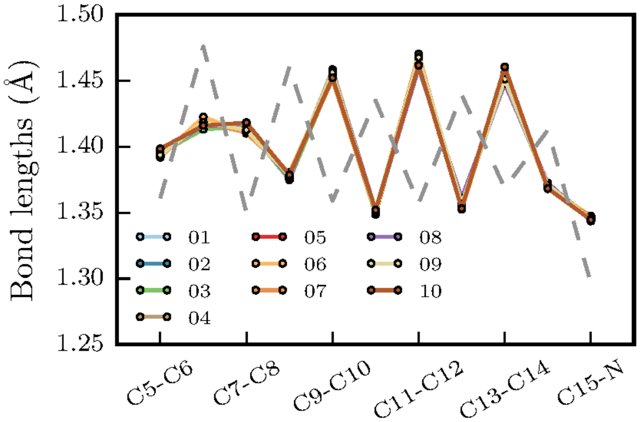

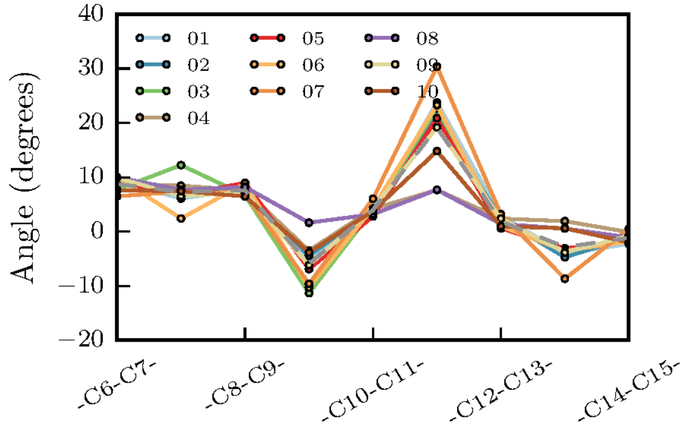

Figure S10. Geometrical parameters obtained after excited state optimizations in M10. Dashed gray lines indicate ground state average geometry. Top: bond lengths; Bottom: deviations from planarity of dihedral angles along the PSB conjugated chain.

Bond relaxation pathways

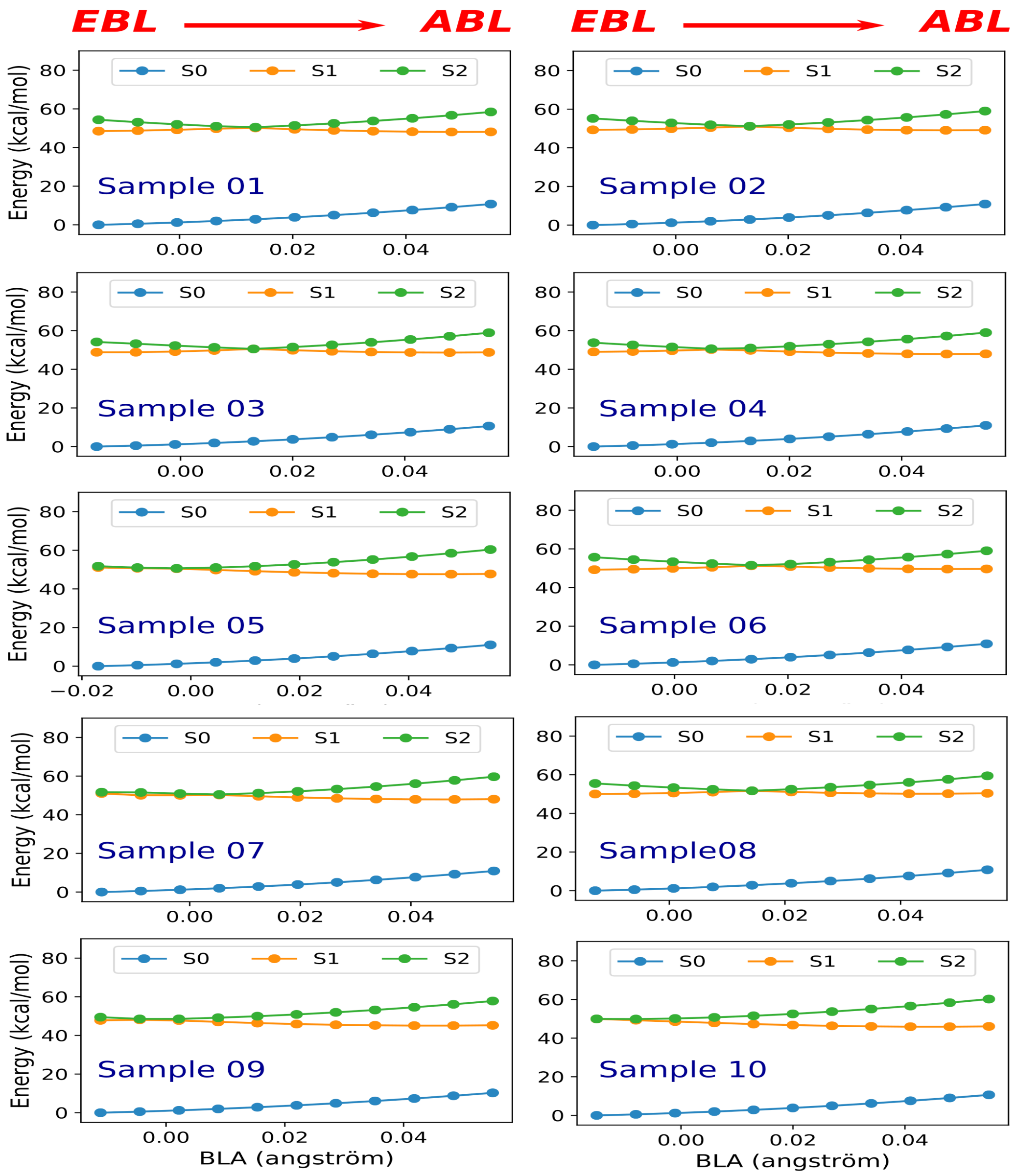

Figure S11. CASPT2/6-31G* potential energy surfaces computed along the C-C stretching mode of the PSB in M4.

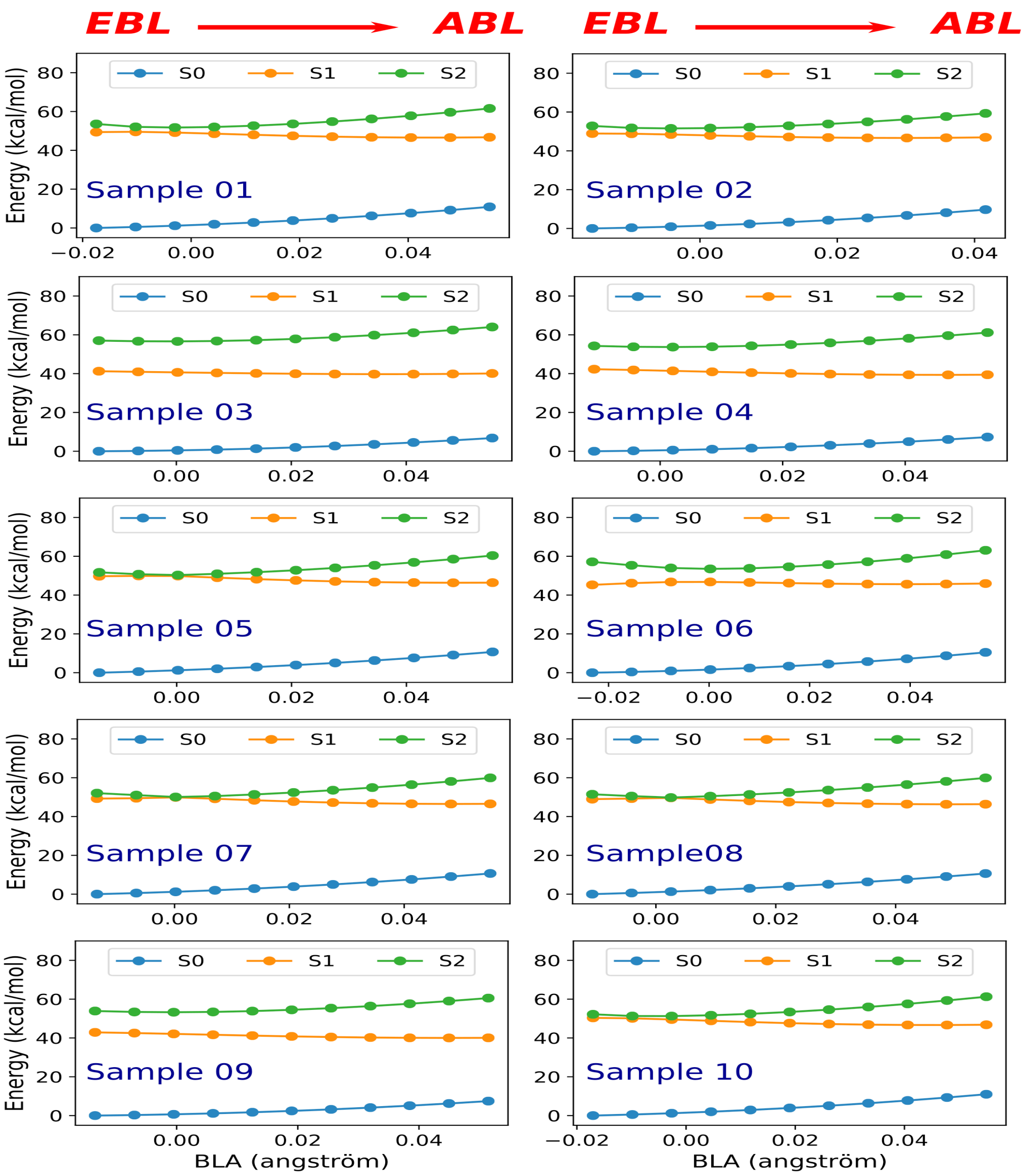

Figure S12. CASPT2/6-31G* potential energy surfaces computed along the C-C stretching mode of the PSB in M8.

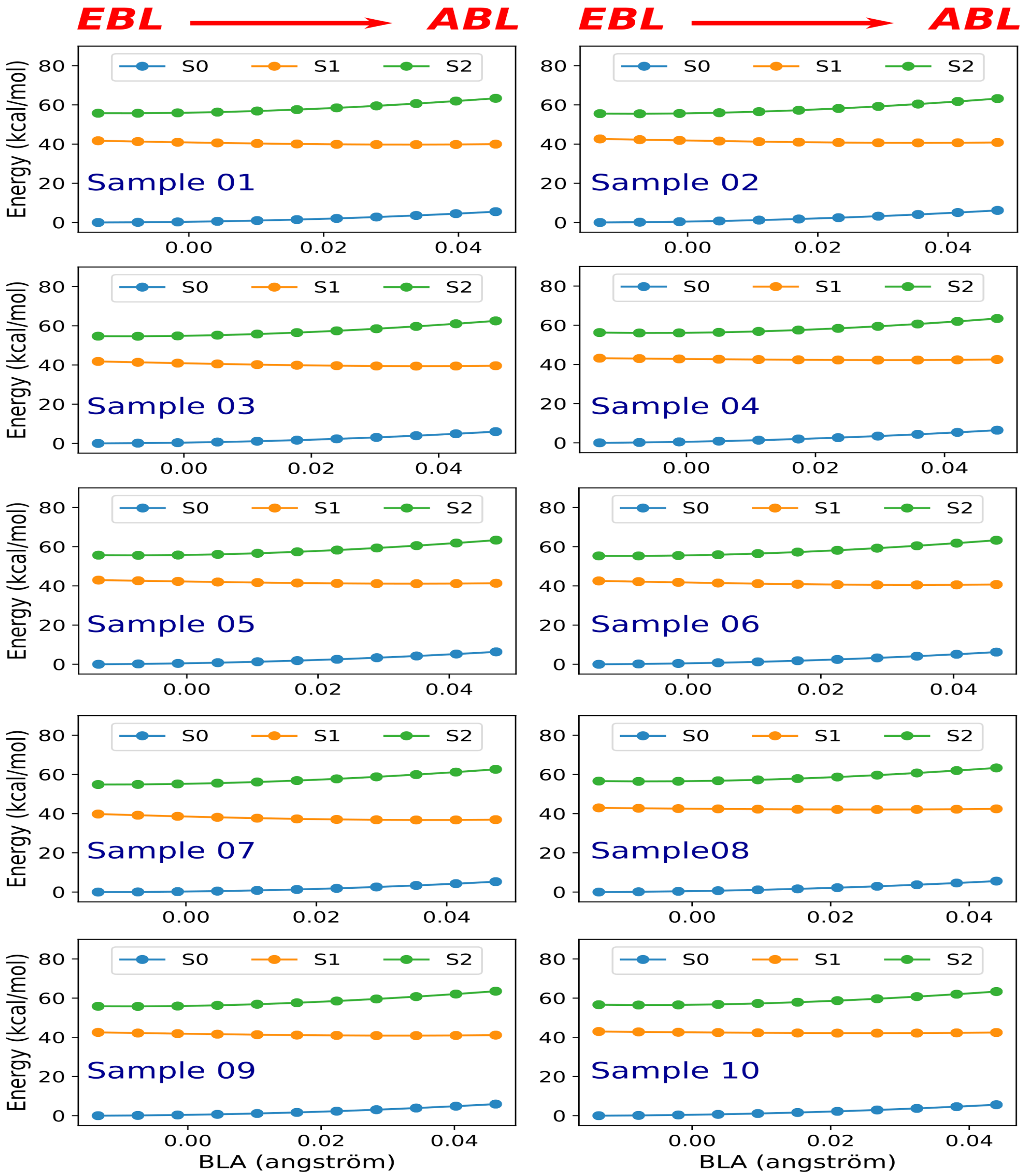

Figure S13. CASPT2/6-31G* potential energy surfaces computed along the C-C stretching mode of the PSB in M10.

Theoretical spectroscopy data

Table S8. Transitions energies (in the 300-800 nm spectral window) and oscillator strengths (only if > 0.1 in absolute value) are reported for GSB and SE (at EBL and ABL geometries) in M4.

Signal type	Osc. Strength	Energy (nm)	Energy (ev)
GSB	1,62	507	2,44
	1,55	507	2,44
	1,74	510	2,43
	1,61	507	2,45
	1,81	506	2,45
	1,65	493	2,52
	1,79	499	2,49
	1,48	480	2,58
	1,68	505	2,46
	1,75	509	2,43
SE (EBL)	1,72	551	2,25
	1,71	548	2,26
	1,86	560	2,21
	1,73	555	2,24
	1,94	536	2,31
	1,65	541	2,29
	1,91	542	2,29
	1,45	533	2,33
	1,53	575	2,16
	1,90	553	2,24
SE (ABL)	1,48	608	2,04
	1,46	610	2,03
	1,65	616	2,01
	1,47	610	2,03
	1,78	603	2,06
	1,38	604	2,05
	1,78	611	2,03
	1,04	605	2,05
	1,28	623	1,99
	1,63	605	2,05

Table S9. Transitions energies (in the 300-800 nm spectral window) and oscillator strengths (only if > 0.1 in absolute value) are reported for ESA (at FC, EBL and ABL geometries) in M4.

ESA	Osc. Strength	Energy (nm)	Energy (ev)
$(FC)_1$	-0,69	403	3,07
	-0,55	434	2,86
	-0,79	410	3,02
	-0,55	431	2,88
	-1,16	413	3,00
	-0,53	445	2,79
	-1,24	409	3,03
$(FC)_2$	-0,27	340	3,65
	-0,20	349	3,56
	-0,17	318	3,90
	-0,31	340	3,65
$(FC)_3$	-0,15	714	1,74
	-0,11	723	1,71
	-0,16	763	1,62
$(ABL)_1$	-0,51	330	3,75
	-0,49	329	3,77
	-0,26	326	3,81
	-0,49	330	3,76
	-0,20	328	3,78
	-0,32	325	3,82
	-0,16	347	3,57
	-0,13	330	3,76
	-0,22	309	4,01
	-0,27	319	3,89
$(ABL)_2$	-0,43	762	1,63
	-0,35	751	1,65
	-0,33	748	1,66
	-0,37	765	1,62
	-0,36	762	1,63
	-0,40	819	1,51
	-0,44	834	1,49
	-0,51	846	1,47
	-0,42	825	1,50
$(ABL)_3$	-0,37	386	3,21
	-0,41	389	3,18
	-0,35	383	3,24
	-0,25	389	3,18
$(ABL)_4$	-0,25	522	2,38
	-0,27	527	2,35
	-0,40	499	2,49
	-0,29	523	2,37
	-0,42	484	2,56
$(ABL)_5$	-0,34	481	2,58

	-0,54	494	2,51
	-0,53	492	2,52
	-0,52	490	2,53
	-0,50	493	2,52
$(EBL)_1$	-0,20	497	2,50
	-0,22	513	2,42
$(EBL)_2$	-0,13	668	1,86
	-0,13	664	1,87
$(EBL)_3$	-0,20	439	2,82
	-0,23	454	2,73

Table S10. Transitions energies (in the 300-800 nm spectral window) and oscillator strengths (only if > 0.1 in absolute value) are reported for GSB and SE (at EBL and ABL geometries) in M8, for the M8' configurations.

Signal type	Osc. Strength	Energy (nm)	Energy (ev)
GSB	1,03	605	2,05
	0,92	574	2,16
	0,98	565	2,19
SE (ABL)	1,45	859	1,44
	1,12	890	1,39
	1,04	878	1,41
SE (EBL)	1,63	705	1,76
	1,32	692	1,79
	1,27	683	1,82

Table S11. Transitions energies (in the 300-800 nm spectral window) and oscillator strengths (only if > 0.1 in absolute value) are reported for ESA (at FC, EBL and ABL geometries) in M8, for M8' configurations.

ESA	Osc. Strength	Energy (nm)	Energy (ev)
$(FC)_1$	-0,49	553	2,24
	-0,58	552	2,25
	-0,54	558	2,22
$(FC)_2$	-0,08	416	2,98
	-0,11	420	2,96
	-0,08	435	2,85
$(FC)_3$	-0,41	630	1,97
	-0,36	589	2,10
	-0,40	655	1,89
$(ABL)_1$	-0,24	394	3,15
	-0,38	428	2,90
	-0,38	428	2,90
$(ABL)_2$	-0,61	522	2,38
	-0,49	574	2,16
	-0,51	568	2,18
$(ABL)_3$	-0,20	726	1,71
	-0,20	747	1,66
	-0,20	690	1,80
$(ABL)_4$	-0,15	489	2,54
	-0,15	478	2,59
	-0,38	486	2,55
$(EBL)_1$	-0,31	505	2,45
	-0,28	502	2,47
$(EBL)_2$	-0,28	653	1,90
	-0,30	671	1,85
	-1,10	696	1,78
$(EBL)_3$	-0,30	491	2,52
	-0,29	489	2,54
	-0,13	495	2,51
$(EBL)_4$	-0,39	572	2,17
	-0,36	573	2,16

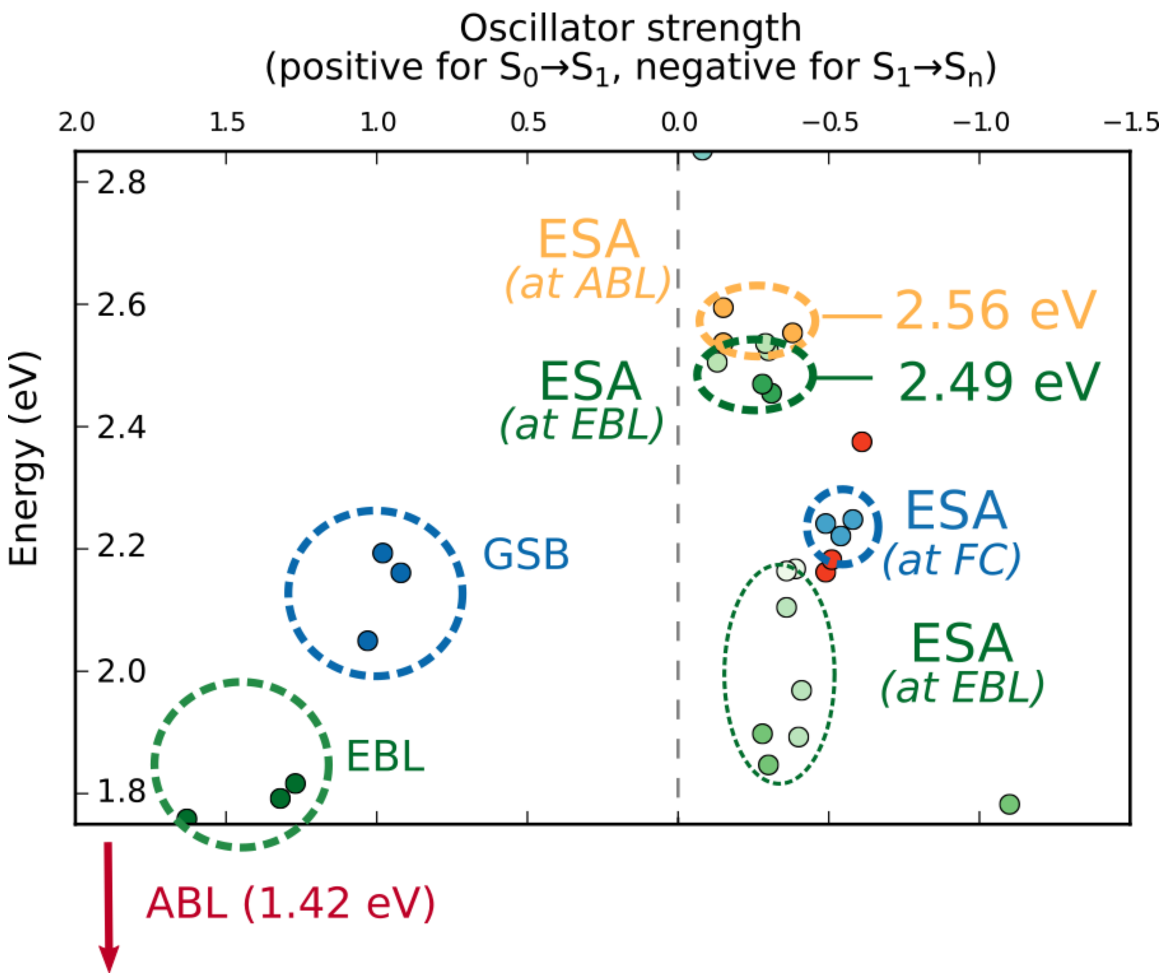

Figure S14. Vertical excitation energies computed at the CASPT2 level (with ANO-S basis set) on top of S_0 minimum (FC, in blue), EBL (green) and ABL (red and orange) geometries of M8' configurations of M8. Computed excited state absorptions (ESA) have negative oscillator strengths to be distinguished from GS bleaching (GSB) and stimulated emissions (SE) signals with positive oscillator strengths.

Table S12. Transitions energies (in the 300-800 nm spectral window) and oscillator strengths (only if > 0.1 in absolute value) are reported for GSB and SE (at EBL and ABL geometries) in M10.

Signal type	Osc. Strength	Energy (nm)	Energy (ev)
GSB	1,28	628	1,97
	1,20	617	2,01
	1,19	612	2,02
	1,17	631	1,97
	1,19	614	2,02
	1,15	609	2,04
	1,18	617	2,01
	1,30	643	1,93
	1,24	622	1,99
	1,02	591	2,10
SE (EBL)	1,69	698	1,78
	1,64	683	1,82
	1,58	696	1,78
	1,78	674	1,84
	1,63	678	1,83
	1,58	685	1,81
	1,46	731	1,70
	1,86	677	1,83
	1,72	685	1,81
	1,48	645	1,92
SE (ABL)	1,43	833	1,49
	1,37	827	1,50
	1,33	852	1,46
	1,51	794	1,56
	1,36	820	1,51
	1,32	832	1,49
	1,28	905	1,37
	1,59	779	1,59
	1,44	816	1,52
	1,15	801	1,55

Table S13. Transitions energies (in the 300-800 nm spectral window) and oscillator strengths (only if > 0.1 in absolute value) are reported for ESA (at FC, EBL and ABL) in M10.

ESA	Osc. Strength	Energy (nm)	Energy (ev)
$(FC)_1$	-1,12	608	2,04
	-1,14	612	2,02
	-1,14	620	2,00
	-1,14	618	2,01
	-1,16	632	1,96
	-1,18	635	1,95
	-1,19	634	1,96
	-1,16	636	1,95
	-1,20	659	1,88
	-1,17	633	1,96
$(ABL)_1$	-0,60	541	2,29
	-0,63	549	2,26
	-0,87	583	2,13
	-0,83	570	2,18
	-0,87	567	2,19
$(ABL)_2$	-0,28	518	2,39
	-0,29	519	2,39
	-0,33	523	2,37
	-0,50	540	2,30
	-0,48	539	2,30
	-0,51	545	2,28
	-0,61	550	2,26
	-0,65	581	2,13
	-0,70	559	2,22
$(ABL)_3$	-0,60	550	2,26
	-0,23	515	2,41
	-0,28	498	2,49
	-0,49	537	2,31
$(EBL)_1$	-0,33	571	2,17
	-0,94	645	1,92
	-1,03	665	1,86
	-1,11	685	1,81
	-1,13	686	1,81
	-1,15	691	1,79
	-1,23	691	1,80
	-1,23	697	1,78
	-0,58	681	1,82
	-1,25	707	1,75
$(EBL)_2$	-0,13	477	2,60
	-0,15	508	2,44
	-0,10	508	2,44
	-0,11	511	2,43
	-0,10	509	2,43
	-0,09	508	2,44
$(EBL)_3$	-0,29	570	2,18
	-0,18	553	2,24
	-0,46	584	2,12
	-0,14	547	2,27

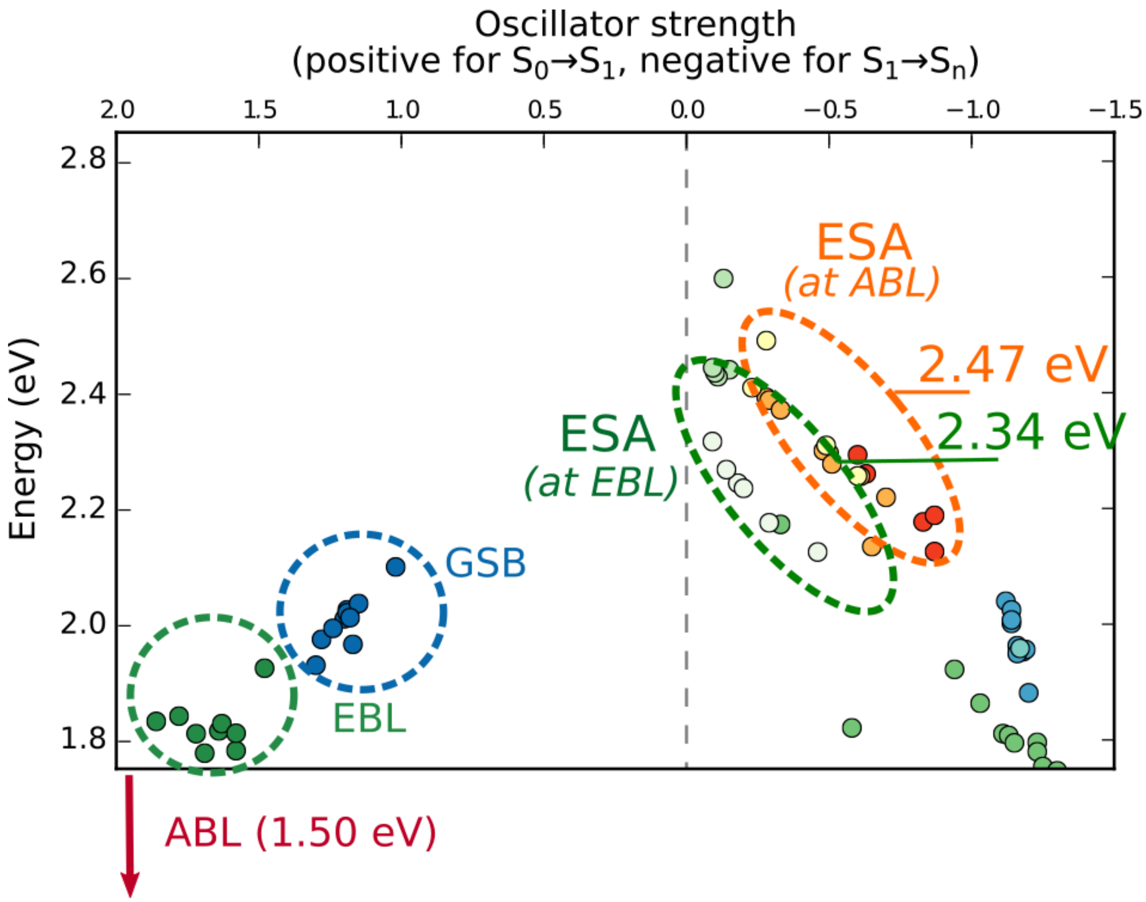

Figure S15. Vertical excitation energies computed at the CASPT2 level (with ANO-S basis set) on top of S_0 minimum (FC, in blue), EBL (green) and ABL (red and orange) geometries of M10. Computed excited state absorptions (ESA) have negative oscillator strengths to be distinguished from GS bleaching (GSB) and stimulated emissions (SE) signals with positive oscillator strengths.

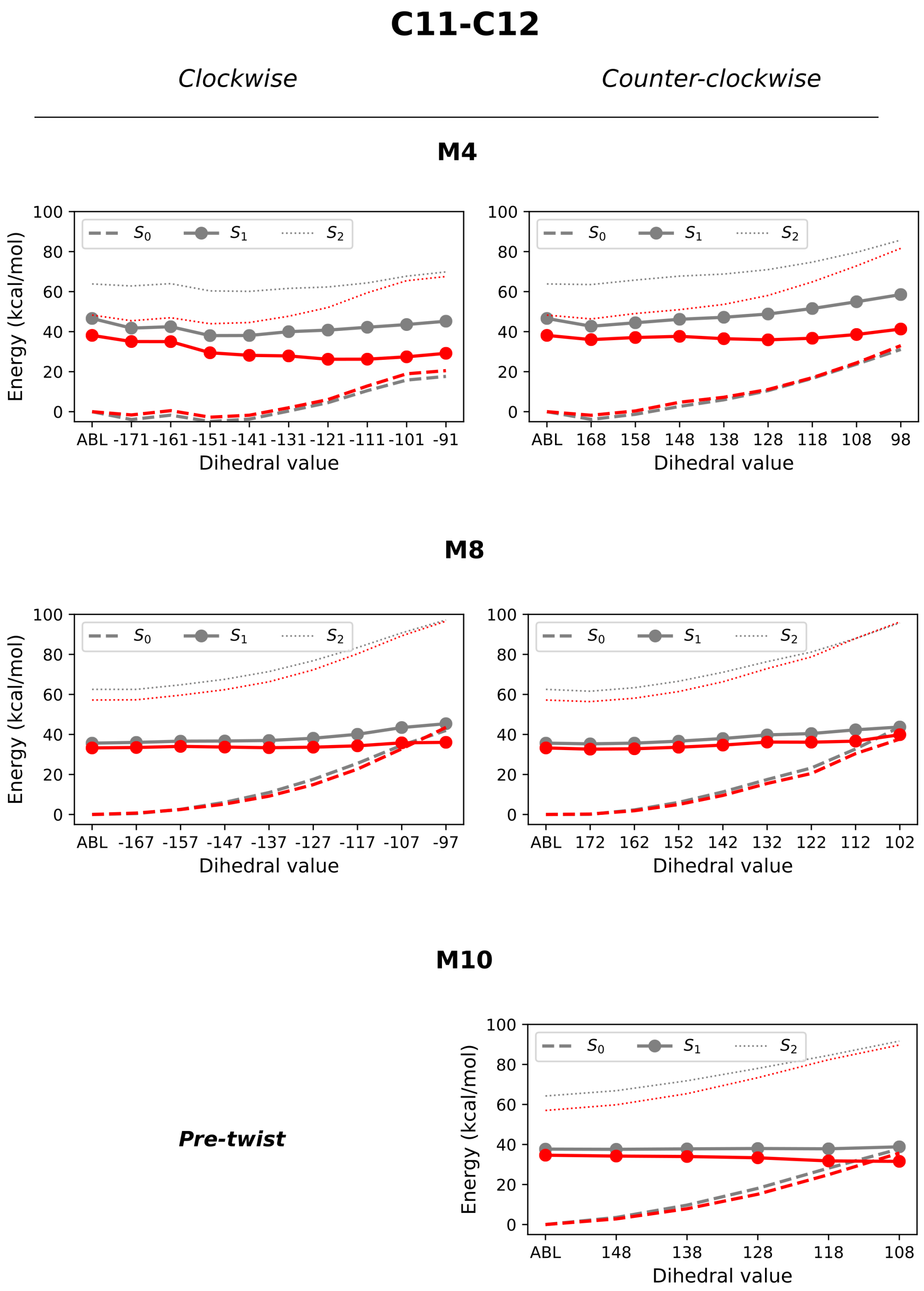

Figure S16. Constrained scans for the rotation around the C11-C12 bond for representative configurations of M4, M8 and M10 proteins. The PES were obtained at the CASPT2 (red) and CASSCF (gray) levels, with the 6-31G* basis set. If the dihedral angle around C11-C12 is pre-twisted in one direction (e.g. counter-clockwise for M10) the opposite direction is not considered.

C13-C14

Clockwise | *Counter-clockwise*

M4

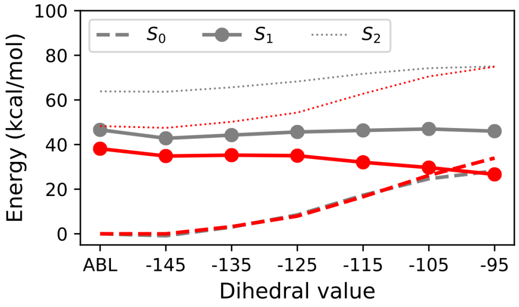

Pre-twist

M8

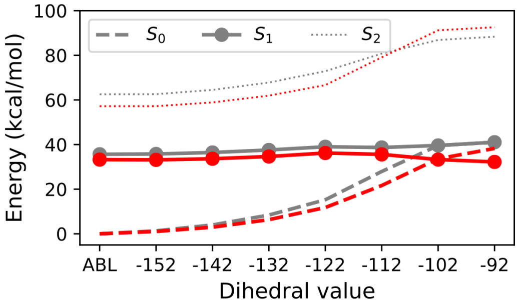

Pre-twist

M10

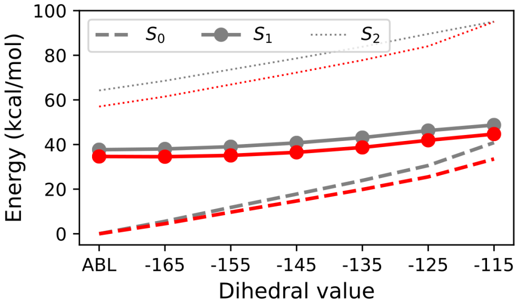

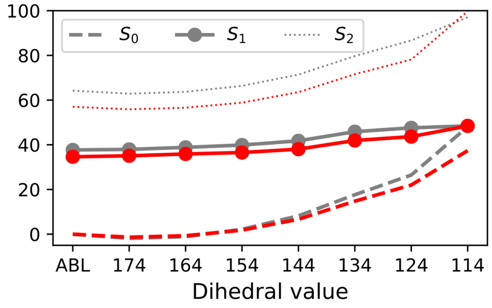

Figure S17. Constrained scans for the rotation around the C13-C14 bond for representative configurations of M4, M8 and M10 proteins. The PES were obtained at the CASPT2 (red) and CASSCF (gray) levels, with the 6-31G* basis set. If the dihedral angle around C11-C12 is pre-twisted in one direction (e.g. counter-clockwise for M10) the opposite direction is not considered.

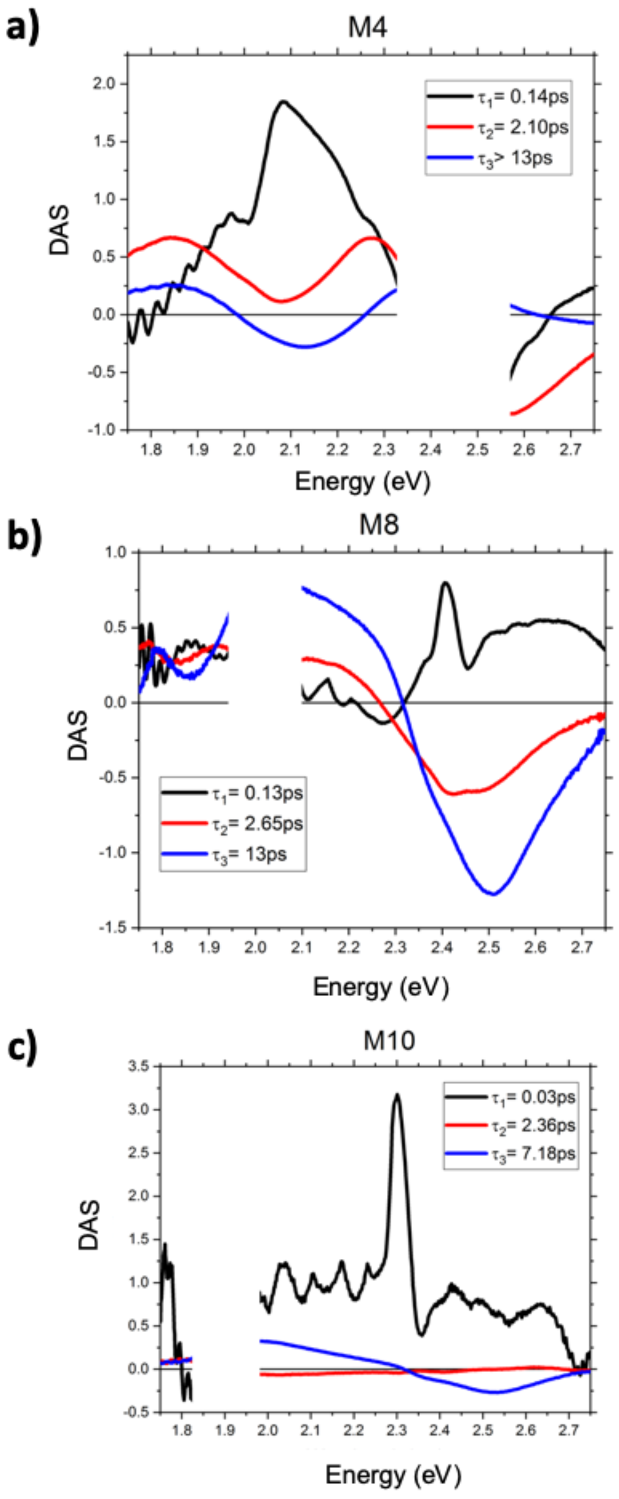

Figure S18. Experimental decay associated spectra (DAS) for the M4, M8 and M10 proteins and corresponding time constants. Note that the DAS do not fully capture the instantaneous signals observed for M8 and M10 and corresponding to stimulated Raman scattering from the aqueous solvent.

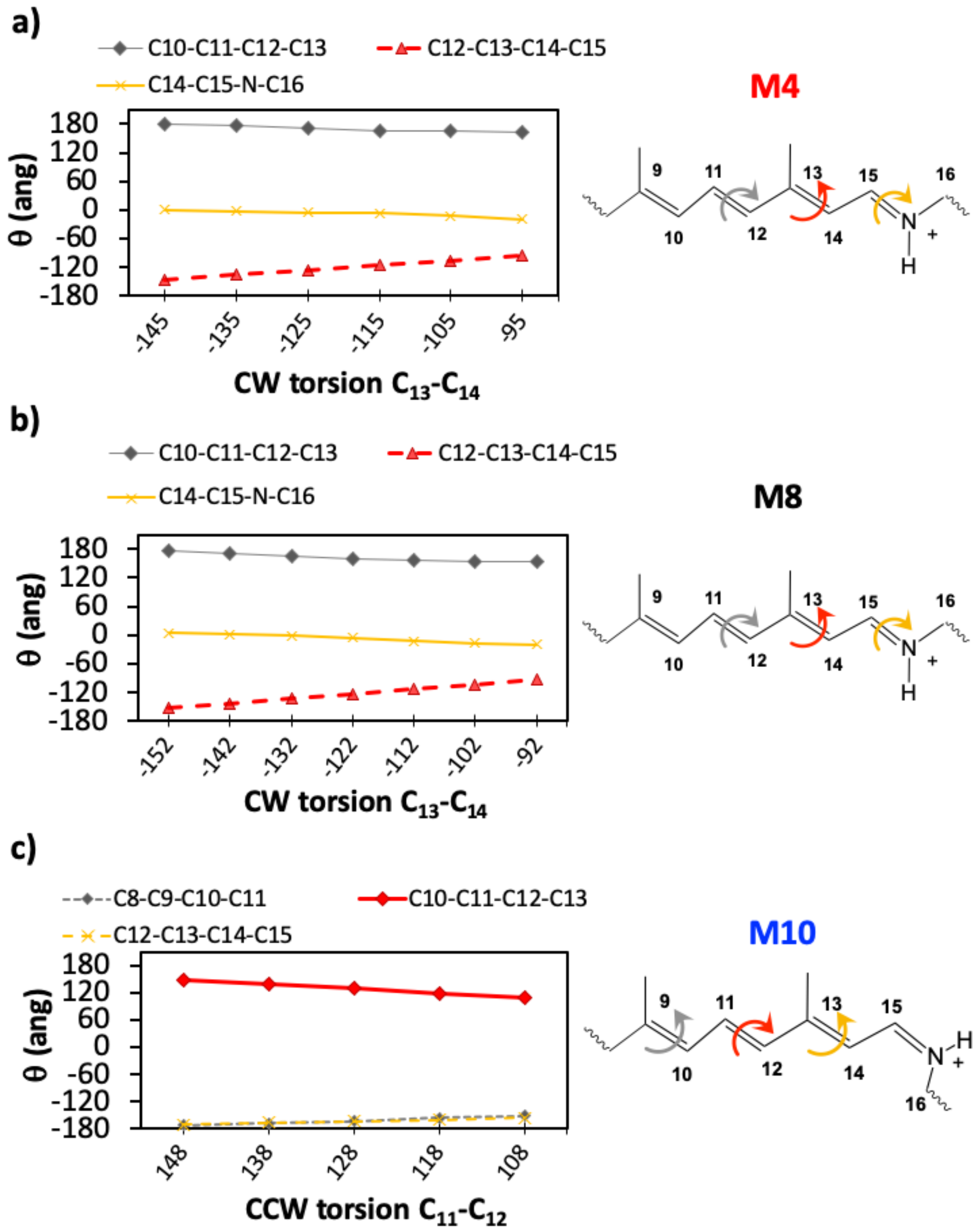

Figure S19. Dihedral angles variations along the most relevant photoisomerization MEPs of (a) M4, (b) M8 and (c) M10 proteins and schematic representation of the bicycle pedal mechanisms.
